# Label-Free SERS of Urine Components: A Powerful Tool for Discriminating Renal Cell Carcinoma through Multivariate Analysis and Machine Learning Techniques

**DOI:** 10.3390/ijms25073891

**Published:** 2024-03-31

**Authors:** Bogdan Adrian Buhas, Valentin Toma, Jean-Baptiste Beauval, Iulia Andras, Răzvan Couți, Lucia Ana-Maria Muntean, Radu-Tudor Coman, Teodor Andrei Maghiar, Rareș-Ionuț Știufiuc, Constantin Mihai Lucaciu, Nicolae Crisan

**Affiliations:** 1 Department of Urology, La Croix du Sud Hospital, 52 Chemin de Ribaute St., 31130 Quint Fonsegrives, France; buhasbogdan@yahoo.co.uk (B.A.B.); jbbeauval@gmail.com (J.-B.B.); 2 Department of Urology, Clinical Municipal Hospital, 11 Tabacarilor St., 400139 Cluj-Napoca, Romania; dr.iuliaandras@gmail.com (I.A.); drnicolaecrisan@gmail.com (N.C.); 3 Faculty of Medicine and Pharmacy, University of Oradea, 1 Universitatii St., 410087 Oradea, Romania; razvan.couti@gmail.com (R.C.); teodormaghiar@yahoo.com (T.A.M.); 4 Department of Nanobiophysics, MedFuture Research Center for Advanced Medicine, “Iuliu Hatieganu” University of Medicine and Pharmacy, 4-6 Pasteur St., 400337 Cluj-Napoca, Romania; valentin.toma@umfcluj.ro; 5 Faculty of Medicine, “Iuliu Hatieganu” University of Medicine and Pharmacy, 8 Victor Babes St., 400347 Cluj-Napoca, Romania; 6 Department of Medical Education, “Iuliu Hatieganu” University of Medicine and Pharmacy, 8 Victor Babes St., 400347 Cluj-Napoca, Romania; ana.muntean@umfcluj.ro; 7 Department of Pharmaceutical Physics–Biophysics, Faculty of Pharmacy, “Iuliu Hatieganu” University of Medicine and Pharmacy, 6 Pasteur St., 400349 Cluj-Napoca, Romania; 8 Nanotechnology Laboratory, TRANSCEND Research Center, Regional Institute of Oncology, 700483 Iași, Romania

**Keywords:** surface-enhanced Raman scattering, silver nanoparticles, urine, kidney cancer, multivariate analysis, machine learning

## Abstract

The advent of Surface-Enhanced Raman Scattering (SERS) has enabled the exploration and detection of small molecules, particularly in biological fluids such as serum, blood plasma, urine, saliva, and tears. SERS has been proposed as a simple diagnostic technique for various diseases, including cancer. Renal cell carcinoma (RCC) ranks as the sixth most commonly diagnosed cancer in men and is often asymptomatic, with detection occurring incidentally. The onset of symptoms typically aligns with advanced disease, aggressive histology, and unfavorable prognosis, and therefore new methods for an early diagnosis are needed. In this study, we investigated the utility of label-free SERS in urine, coupled with two multivariate analysis approaches: Principal Component Analysis combined with Linear Discriminant Analysis (PCA-LDA) and Support Vector Machine (SVM), to discriminate between 50 RCC patients and 44 healthy donors. Employing LDA-PCA, we achieved a discrimination accuracy of 100% using 13 principal components, and an 88% accuracy in discriminating between different RCC stages. The SVM approach yielded a training accuracy of 100%, a validation accuracy of 99% for discriminating between RCC and controls, and an 80% accuracy for discriminating between stages. The comparative analysis of raw and normalized SERS spectral data shows that while raw data disclose relative concentration variations in urine metabolites between the two classes, the normalization of spectral data significantly improves the accuracy of discrimination. Moreover, the selection of principal components with markedly distinct scores between the two classes serves to alleviate overfitting risks and reduces the number of components employed for discrimination. We obtained the accuracy of the discrimination between the RCC patients cases and healthy donors of 90% for three PCs and a linear discrimination function, and a 88% accuracy of discrimination between stages using six PCs, mitigating practically the risk of overfitting and increasing the robustness of our analysis. Our findings underscore the potential of label-free SERS of urine in conjunction with chemometrics for non-invasive and early RCC detection.

## 1. Introduction

According to the Global Cancer Observatory, kidney cancer is the ninth most common cancer among men and the fourteenth most common among women, with an estimated 431,288 new cases in 2020 globally [1]. Generally, during the last two decades until recently, there has been an annual increase of about 2% in incidence both worldwide and in Europe. In 2022, worldwide mortality from RCC was 179,368 deaths (115,600 men and 63,768 women), with a calculated global age-standardized rate of 1.8/100,000 [2]. There is a 1.5–2.0:1 predominance in men over women with a higher incidence in the older population [2,3,4].

RCC is the most common solid lesion within the kidney and accounts for approximately a significant majority (90%) of kidney cancer cases, predominantly including clear cell RCC (ccRCC; 70%), papillary RCC (pRCC; 10–15%), and chromophobe RCC (chRCC; 5%) [5]. RCC represents around 3% of all cancers, with the highest incidence occurring in Western countries [3,6].

Many renal masses remain asymptomatic until the late disease stages. At the same time, the majority of RCCs are detected incidentally by non-invasive images investigating various non-specific symptoms for other abdominal diseases [7]. The once classical triad of hematuria, pain, and abdominal mass is now recognized to be rare, and symptoms, if present at all, can be vague, non-specific, and delayed in onset. While early diagnosis is recognized to be key in achieving optimal outcomes, many patients still present with advanced disease [8,9,10].

In recent years, there has been a growing interest from both patients and clinicians in RCC screening programs; however, there is a relative lack of studies reporting the efficacy, cost-effectiveness, and optimal modality for RCC screening [11,12,13]. 

Numerous molecular markers, including carbonic anhydrase IX (CaIX), Vascular endothelial growth factor (VEGF), Hypoxia-inducible factor (HIF), Ki67 (proliferation), p53, p21 [14], PTEN (phosphatase and Tensin Homolog) cell cycle [15], E-cadherin, osteopontin [16], CD44 (cell adhesion) [17,18], CXCR4 [19], PD-L1 [20], miRNA, SNPs, gene mutations, and gene methylations have undergone investigation [21]; however, the outcomes have not demonstrated promising results.

Kidney-injury molecule-1 (KIM-1) in both urinary and plasma samples has emerged as a prospective diagnostic and prognostic indicator. Elevated concentrations of KIM-1 have been linked to a reduced life expectancy and demonstrated the ability to anticipate the onset of RCC up to five years before formal diagnosis [22]. The activity of the BAP1 and PBRM1 genes, located on chromosome 3p within a region deleted in over 90% of ccRCC, has demonstrated their status as autonomous prognostic factors for tumor recurrence [23,24,25]. This implies that patients with BAP1-mutant tumors may experience less favorable outcomes compared to those with PBRM1-mutant tumors [24]. Nevertheless, it is important to note that these signatures have not undergone validation by independent researchers, and as a result, their routine application in clinical practice is not recommended.

In the last decades, vibrational spectroscopy techniques such as infrared and Raman spectroscopy, through many proof-of-concept studies, have been revealed as methods with huge potential in the field of medical diagnosis [26]. The molecular vibration spectra of biological tissues or liquids harbor crucial information about their biochemical composition, potentially enabling the discrimination of various pathological conditions, including cancer. The development of the Surface-Enhanced Raman Scattering (SERS) technique, which uses either gold or silver nanoparticles to amplify the Raman signals of molecules that attach to metal surfaces, opened the way for investigating and detecting small molecules, especially in biological fluids within the so-called liquid biopsies. The SERS investigation of biological liquids such as serum or blood plasma, urine, saliva, and tears presents major advantages from the point of view of their cost-effectiveness, non-invasive collection, the small amounts of samples required, the non-destructive nature of the investigation, and the openness of the patients. It has been proposed as a straightforward technique for diagnosing various diseases, including cancer [27]. SERS spectra are significantly influenced by the affinity between the SERS substrate (gold or silver nanoparticles) and the molecules under investigation and their relative orientation. Despite challenges related to low reproducibility, judicious choices in SERS substrate and appropriate functionalization have achieved unparalleled performances in the detection of biologically relevant molecules [28]. The low reproducibility of spectra is influenced by factors such as the nature of the substrate, sample state (solid/dry or liquid), ionic composition, and wavelength of the Raman excitation laser.

When dealing with biological liquids, SERS spectra of colloids are often dominated by proteins due to nanoparticle opsonization [29]. Detection of lower molecular mass components requires prior deproteinization of the sample, either through filtration or chemical means. As an alternative, solid plasmonic substrates could be used to provide spectra dominated by lower molecular mass compounds [30]. In previous reports, it was demonstrated that reproducible SERS spectra, obtained through a solid plasmonic SERS substrate, allowed for a possibility of discrimination of breast cancer patients through label-free SERS of blood plasma, combined with multivariate analysis [31]. Also, we recently proved that label-free SERS of blood serum analyzed either through Principal Component Analysis combined with Linear Discriminate Analysis (PCA-LDA) or through Support Vector Machine (SVM) could discriminate with high accuracy RCC patients from apparently healthy controls [32]. 

In the present work, we extended our research towards the label-free SERS profiling of urine for accurate discrimination of RCC, demonstrating that urine SERS might produce comparable figures of merit as compared to blood serum. 

By using urine samples from 50 RCC male patients and 44 controls and employing simple statistical tests, we enhanced the assignment of major vibrational bands to gain deeper insights into factors influencing discrimination accuracy. Correctly assigning vibration bands contributes not only to accurate disease discrimination, but also aids in understanding diseases at a biochemical level, suggesting avenues for further research and study.

Both Principal Component Analysis combined with PCA-LDA and SVM techniques were used to analyze the experimental data. We illustrate how different parameters in statistical analysis can enhance the accuracy of SERS discrimination of RCC patients from healthy donors. While multivariate analysis and machine learning algorithms often achieve high discrimination accuracy, precise interpretation of label-free SERS data and understanding the molecular origin of each band from a biochemical perspective enhance result credibility. 

## 2. Results and Discussion

### 2.1. Surface-Enhanced Raman Scattering (SERS) of Urine Samples and Correlations between the Surface-Enhanced Raman Scattering (SERS) Vibrational Bands

**Scheme 1:**
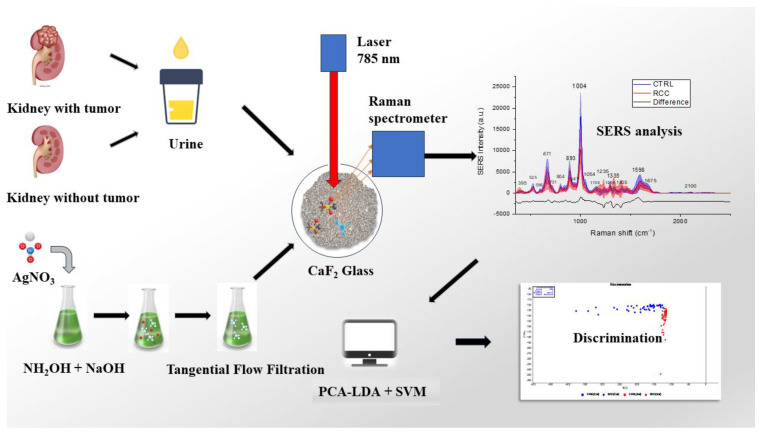
Schematic procedure of the experimental workflow.

The overall procedure for the SERS substrate preparation, sample deposition, and data acquisition is depicted in Scheme 1 and described in detail in Section 3. Each urine sample was placed onto the substrate, and SERS spectra were systematically recorded at 50 distinct points across the dried droplet using the Raman spectrometer in mapping mode. To ensure robustness, two sets of maps, each consisting of 50 points, were averaged, resulting in each sample spectrum representing the mean of 100 SERS spectra. These mapping points were positioned approximately 50 µm from the edge of the dried droplet within regions of uniform thickness.

**Figure 1:**
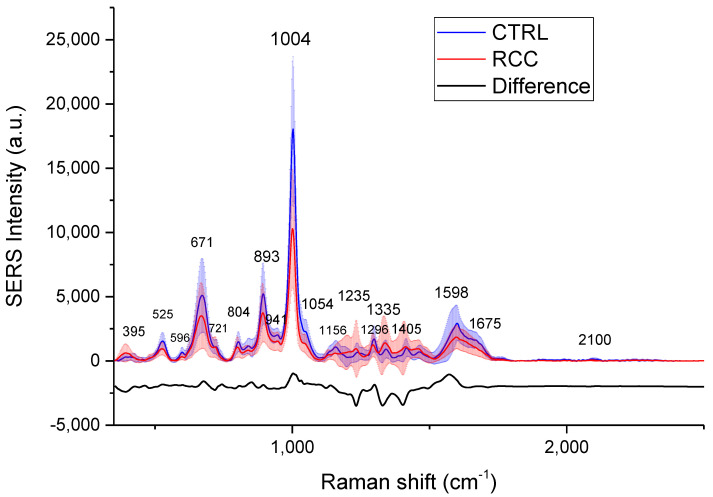
Mean SERS spectra of urine samples for controls (blue) and RCC (red) with the main vibration peaks, and the difference between the two mean spectra (black). Dashed areas represent the standard deviations.

Figure 1 displays the mean spectra recorded for cancer samples (depicted in red) and controls (depicted in blue) and the difference spectrum (black), highlighting the major vibrational peaks and their corresponding Raman shifts, recorded using an excitation wavelength of 785 nm. The SERS spectra of all samples from both control subjects and RCC patients are provided in Figure S1. Additionally, the mean SERS intensities for the two maps were computed, revealing a substantial overlap between the two datasets (Figure S2A), confirming the quality of the SERS substrate. There is only a slight increase in the SERS intensities measured on the second map. However, these differences in the two maps disappeared and there is almost a perfect match (Figure S2B) between the two sets of data after the spectra were normalized to the maximum peak intensity (at 1004 cm^−1^). 

The major vibrational peaks, their corresponding Raman shifts, and their assignments, drawn from literature data and proposed in this study, are outlined in Table 1. It is worth noting that slight variations exist in the wavenumbers of reported SERS peaks in the literature. For this reason, in the second column of Table 1, we offer ranges instead of fixed wavenumbers to account for these differences.

The SERS spectra of urine reported so far in the literature present a much larger variability as compared to those of blood plasma or serum. This variability was explained by the different manners in which the samples were prepared: with or without centrifugation, measurement of the sediment or the supernatant, the nature of the substrate (either colloid or solid), the state of the sample (liquid or dried), the pH, the excitation wavelength, etc. 

The assignment of SERS vibrational bands in urine lacks consensus within the SERS community. Table 1 clearly illustrates that different molecular species and sometimes even different vibration modes are assigned to the same experimentally observed Raman shift wavenumber. These inconsistencies primarily arise from assigning SERS bands to wavenumbers observed in the Raman spectra of biological molecules without clear experimental evidence of their presence or relative concentration in urine. A lack of comprehensive databases containing well-characterized SERS spectra of urine components makes identifying and assigning specific vibrational bands challenging. The absence of standardized reference data hinders the comparison and validation of results across studies. Only a few papers compare the SERS signals of urine with those of relevant molecules obtained under the same experimental conditions. These conditions include the use of the same substrate, excitation wavelength, and ionic composition of the medium, among others. Our tentative assignments are based on these references together with combined experimental and theoretical papers related to SERS of urea [44] and creatinine [45], and the correlations between the SERS peaks in our study, as it will be detailed further.

Urine is a complex fluid containing urea as a major organic compound (10–35 g/day per capita) and to a smaller extent creatinine (1–1.8 g/day per capita), uric acid (0.25–0.80 g/day per capita), and creatine (0–0.15 g/day per capita) [58]. Other urine components are phosphate, sodium, potassium, and ammonia. The protein concentration in urine is typically low, the urine is considered protein-free or contains only trace amounts of protein (<0.15 g/day per capita), higher levels of protein (mainly albumin) in the urine, known as proteinuria, being observed in kidney diseases or other medical conditions, usually by measuring the protein to creatinine ratio [59].

In the label-free SERS approach, the spectra of biological liquids strongly depend mainly on molecules affinity for the plasmonic substrate and, to a lesser extent, on their relative concentration. While in the case of colloidal silver and gold nanoparticles the nanoparticles will be surrounded by proteins that will dominate the SERS, in the case of solid plasmonic substrates with a near-infrared excitation wavelength of 785 nm, the most prominent bands observed in SERS mainly correspond to lower molecular mass molecules, even in the case of highly concentrated biofluids, as we recently demonstrated in the case of blood serum [32]. The fact that the solid substrates amplify mostly the Raman signal of small molecules was first observed in a pioneering paper by Premasiri et al. [30]. These authors showed that using a solid substrate the SERS spectrum of blood plasma is dominated by the signature of purinic metabolism (mainly uric acid, hypoxanthine) and by keeping the whole blood for several hours before the separation of the cell, the SERS spectrum of blood serum became dominated by the spectral features of hypoxanthine. Later, a systematic investigation by Bonfacio’s group [29], using ultrafiltration with cut-off filters of 3 kDa and 10 kDa brought supplementary evidence about the major role played by the small molecules in the SERS spectra recorded on blood plasma on solid substrates. In Figure S3, we present the Raman spectrum of a CTRL urine sample together with the corresponding SERS spectrum obtained for the same sample on our solid SERS substrate, demonstrating the enhancement capabilities of the substrate.

As can be seen from Figure 1, the urine SERS obtained in our study is dominated by an intense peak at 1004 cm^−1^ which is associated in most papers with urea, a major component of urine. Indeed, urea is the major component of urine and is characterized by a strong Raman peak at ~1000 cm^−1^ which was proposed to be used for quantifying urea in urine samples by Raman spectroscopy [37,56]. The quantification of urea was proposed also by using SERS on gold nanostars immobilized on a solid surface and functionalized with urease [60]. The SERS spectrum of urea in this study reveals a strong peak at 1010 cm^−1^, whose intensity varies linearly with urea concentration in the range of 2.5–20 mg/mL, which is typically for urea concentration measured in the clinic. In another study, artificial urine SERS spiked with urea, creatinine, and albumin, recorded on gold nanoparticles revealed the most intense peak at 1018 cm^−1^ with intensity increasing linearly with increasing urea concentration in the range of 0.2–1.0 mg/mL [50]. Other assignments of the SERS peak of urine recorded around 1000 cm^−1^ are albumin [42,49,53,54] and phenylalanine [33,41], an amino acid associated with albumin, or its aromatic ring stretching [40]. However, as pointed out earlier, our solid plasmonic platform specifically enhances the Raman signal of small molecules, even in a high protein concentration environment [32]. Moreover, the studies assigning this peak to albumin or its constituents were performed on colloidal SERS platforms sometimes specifically functionalized for the detection of proteins [53]. Therefore, we assigned the 1004 cm^−1^ peak recorded for urine in our experimental conditions to urea. 

Another major SERS peak occurring at 671 cm^−1^ is associated in the majority of the published studies with creatinine [61]. The Raman peak for solid creatinine was recorded at 669 cm^−1^ but in solution and SERS it shifts to higher wavenumbers. Also, the area under this peak was found to vary linearly with the creatinine concentration [61]. Another peak that was used for the creatine concentration determination in water solutions is situated at 836 cm^−1^ [43] both in pure water solutions and in a mixture with ten times higher urea concentrations; however, the intensities of creatinine peaks measured at 602 cm^−1^, 671 cm^−1^, 836 cm^−1^, and 900 cm^−1^ decrease significantly in the presence of urea (detected at 1003 cm^−1^). Also, with decreasing pH the intensity of all SERS peaks assigned to creatinine decreases [61].

Another small molecule with a specific SERS signature usually detected in biological fluid is uric acid. As demonstrated in our previous works [32], there are two intense peaks associated with uric acid in the case of blood serum recorded on our solid plasmonic substrate at 640 cm^−1^ and 1136 cm^−1^, and other medium intensity peaks recorded at 496 cm^−1^, 590 cm^−1^, 813 cm^−1^, and 889 cm^−1^. On the other hand, the uric acid contains three carbonyl (C=O) groups, usually characterized by a stretching vibration above 1700 cm^−1^ readily detected by Raman spectroscopy. In the case of our urine samples from our study, we notice only a peak around 1600 cm^−1^. This might be due either to the fact that the carbonyl groups in uric acids are included in heterocycles which might change the vibration frequency, or to the geometry of uric acid absorption on the substrate, depending on the pH and the protonation/deprotonation status of uric acid. A combined Density Functional Theory (DFT) and experimental study of uric acid SERS on silver colloids [62] assigned an experimental 1607 cm^−1^ and a theoretical 1609 cm^−1^ wavenumber to the C=O stretching in uric acid, supporting the above conclusion. Nevertheless, other molecules or vibration groups (e.g., C=C stretching) might contribute to the SERS signal around 1600 cm^−1^, as discussed below. In the case of our mean SERS spectrum of urine samples (Figure 1), no significant peaks were recorded at 640 cm^−1^ and 1136 cm^−1^. However, if we look at individual samples (Figure S4), we can notice clear peaks situated close to these wavenumbers. Mediated over a large number of samples, these peaks are not well defined being “masked” by the vibrations characteristic for other groups (e.g., the creatinine peak at 671 cm^−1^). Westley et al. [63] reported intense SERS peaks of urine at 640 cm^−1^ and 1134 cm^−1^, which were associated with uric acid and were used to quantify the uric acid in urine by using a standard addition method. However, in the latter study, the plasmonic platform consisted of hydroxylamine-reduced Ag colloids, but the urine samples were pretreated with methanol and afterward vacuum concentrated, which might explain the small differences in the SERS spectra of urine samples in this study, as compared to our study. 

Hu et al. [36] compared the SERS spectrum of both supernatant and sediment of urine samples from bladder cancer patients with that of healthy volunteers, using citrate-reduced silver colloids. The SERS spectra reported in this study especially for the urine supernatants (with slight changes in the sediments) were quite similar to those reported in the present study, with higher intensity of the SERS peak recorded at 1000 cm^−1^ (assigned to urea) and 668 cm^−1^ (assigned to O-P-O vibration in DNA) in healthy volunteers as compared to the peaks recorded at the same wavenumbers for cancer patients. It has to be mentioned that the SERS vibrational band assignments in the above-mentioned study were based on metabolomics studies. Another interesting observation is that in the urine sediments, the SERS peak recorded in supernatants at 668 cm^−1^ is shifted to lower wavenumbers (635 cm^−1^) simialr to the case of methanol-treated and centrifuged samples [63]. In our study, we used clear urine samples without visible suspensions, without further preparation, and therefore the SERS spectra are more similar to the urine supernatant spectra [36]. It is also worth mentioning that the lower-intensity SERS signal at 668 cm^−1^ in the urine supernatant of bladder cancer cases is similar to our results for kidney cancer cases, recorded at a slightly different wavenumber (671 cm^−1^) assigned to creatinine. 

**Figure 2:**
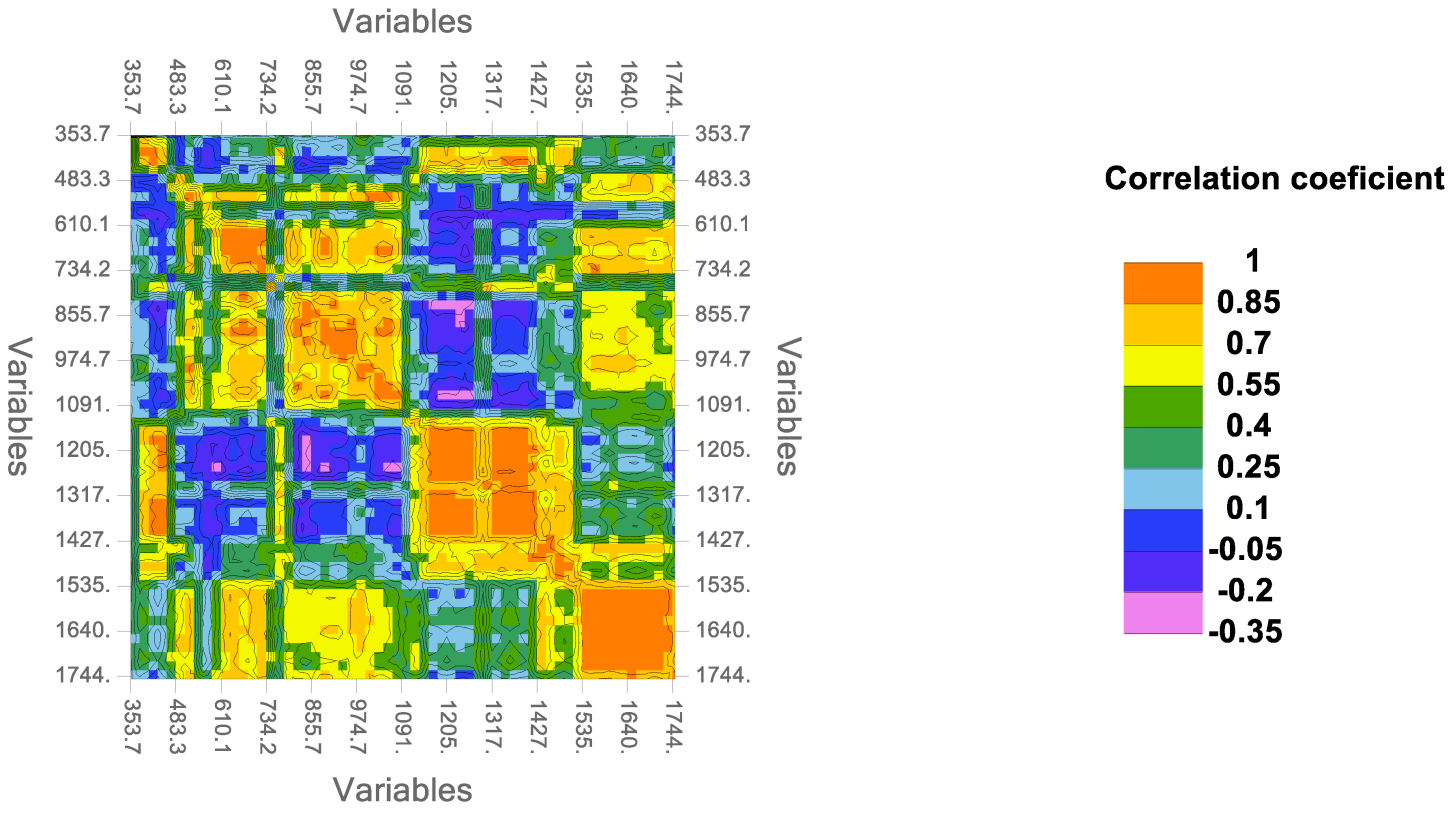
Contour plot of the correlation matrix for the SERS intensities recorded at different wavenumbers (Raman shifts) based on the SERS spectra mediated over all samples. The coefficient of correlation is represented by different colors according to the legend figured on the right of the matrix plot. The variables are the wavenumbers measured in cm^−1^.

To enhance our understanding of the assignment of bands in serum samples, we conducted a linear regression analysis on various SERS peak intensities. This involved calculating the coefficient of correlation for the corresponding linear regressions. The correlation matrix, calculated based on the mean SERS over all samples is presented in Figure 2, as a contour map, where the different colors indicate the correlation coefficients, according to the legend attached to the figure. 

We acknowledge that this type of analysis cannot provide us with clear-cut answers related to the assignments of the SERS peaks. A high value of the correlation coefficient for the linear regression between two SERS intensity peaks suggests that either they belong to the same compound or they belong to different molecules with a similar change in concentration from one sample to the other. Nevertheless, this analytical approach can offer valuable insights into the assignments of the SERS vibrational bands. As a general observation, one can notice high correlation coefficients between the SERS intensities in some ranges of the wavenumbers (e.g., 600–700 cm^−1^, 1535–1750 cm^−1^, 1200–1400 cm^−1^). Focusing on the most intense SERS peaks (not for all wavenumbers as plotted in Figure 2), we present in Figures S4–S6 the regression curves and the corresponding coefficients of determination (R^2^) for the most intense SERS vibrational bands of urine. The 1004 cm^−1^, assigned to urea presents the highest correlation (R^2^~0.84) with the peak at 525 cm^−1^ and a much lower value of the coefficient of determination for the peak at 671 cm^−1^, assigned for creatinine (Figure S4). The latter peak shows high R^2^ values of 0.93 and 0.87 with the peaks at 804 cm^−1^ and 893 cm^−1^, respectively. We also notice a high coefficient of determination among these two latter peaks (0.94). At this point, it is worth mentioning that in the case of serum, the SERS intensity at 640 cm^−1^ and 1135 cm^−1^, assigned to uric acid, correlates with a coefficient of determination higher than 0.9 with the SERS intensities at 812 cm^−1^ and 889 cm^−1^ [32], which are situated very close to the 804 cm^−1^ and 893 cm^−1^ urine peaks recorded in the present study. We therefore cannot exclude a contribution of the uric acid for these peaks, the 640 cm^−1^ being probably masked by the intense creatinine vibration at 670 cm^−1^. No significant correlations with other vibrational bands were observed for the peak (shoulder) at 721 cm^−1^ assigned in the literature to hypoxanthine [34,35] or with the weak peaks recorded at 941 cm^−1^, 1156 cm^−1^, and 1296 cm^−1^ (Figure S5) (the latter two peaks being assigned to cytosine/guanine [40,49]). In the range of 1200–1400 cm^−1^, we recorded several weak SERS peaks, among which between the peaks at 1235 cm^−1^, 1335 cm^−1^, and 1405 cm^−1^ there are high coefficients of determination in the range of 0.91–0.96 (Figure S6), indicating that they might belong to the same types of compounds. As can be noticed from Table 1, the most common assignments for these peaks are to ketone bodies (either hydroxybutyrate or acetoacetate which are usually at equimolar concentrations in urine) and based on the high coefficients of determination calculated between these peaks we considered the same assignment.

Regarding the peaks situated at high wavenumbers (1500–1700 cm^−1^) usually assigned to C=C, C=O, and amide I stretching in proteins, or some amino acids and lipids, we could not find high coefficients of determination with other vibrational bands. At a closer look at this wavenumber range (Figure S7), one can notice that there is a large variability among the samples’ peaks; therefore, it is difficult to assign them to a certain molecule or even a certain type of vibration.

In addition to the assigned Surface-Enhanced Raman Spectroscopy (SERS) spectra corresponding to various metabolites, as demonstrated in prior studies, numerous visible SERS peaks (see Table 1) remain unidentified. These peaks present an opportunity for discovering new biomarkers and warrant further investigation. However, clinical practice often finds that a single biomarker may not achieve optimal diagnostic sensitivity and specificity. Consequently, high-throughput methodologies, including genomics, transcriptomics, proteomics, and metabolomics, have been explored for cancer diagnosis.

### 2.2. Univariate Analysis

**Figure 3:**
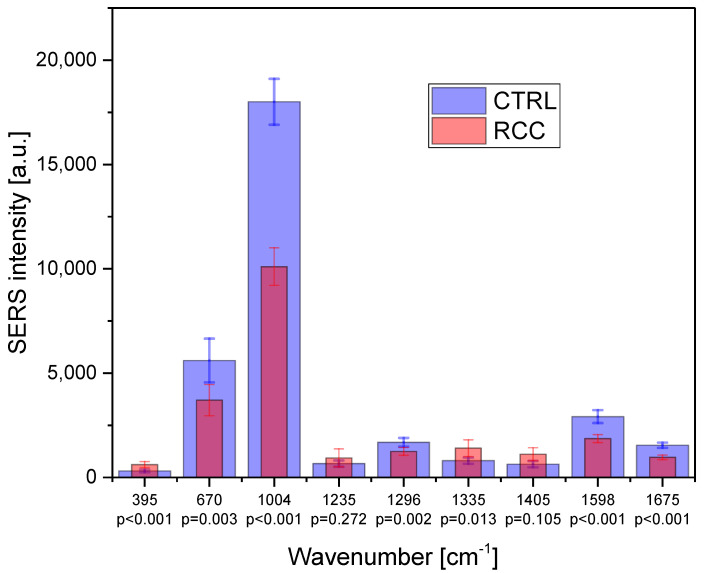
Student’s *t*-test for the most intense vibrational bands of urine. The numbers under the columns are the mean Raman shifts in cm^−1^. Bars are standard deviations. Pearson’s coefficient *p* and the mean number of counts for each peak are highlighted, as well.

To quantitatively assess the differences between RCC patients and healthy donors, we conducted a statistical significance test for the major vibration bands using the Student’s *t*-test. The analysis was carried out with the Unscrambler program, set at a 95% confidence level (α = 0.05). The summarized results of this analysis for the most intense SERS peaks are presented in Figure 3.

The most notable result reveals a highly significant (*p* < 0.001) increase in peak intensity assigned to urea at 1004 cm^−1^ in healthy donors compared to RCC cases. A similar pattern was observed for the peak at 525 cm^−1^, which shows a strong correlation with the 1004 cm^−1^ peak. The same pattern was observed for the 671 cm^−1^ peak assigned to creatinine and exhibited significantly higher SERS intensity in healthy donors cases, along with the peaks at 596 cm^−1^, 804 cm^−1^, and 893 cm^−1^ which demonstrated a high correlation with the 671 cm^−1^ peak. Additionally, a comparable trend was identified for the peaks at 1054 cm^−1^, 1156 cm^−1^, 1296 cm^−1^, 1598 cm^−1^, and 1675 cm^−1^. A reverse scenario, featuring highly significant intense peaks in samples from RCC patients, was observed at 390 cm^−1^ and 1355 cm^−1^. No statistically significant differences in SERS intensity signals were detected at 721 cm^−1^, 1235 cm^−1^, and 1405 cm^−1^. 

At this point, it has to be mentioned that the same significantly higher intensity SERS peaks at around 1000 cm^−1^ (assigned to urea), and at 668 cm^−1^ (assigned to O-P-O in DNA) were recorded for the urine of controls as compared to bladder cancer patients [36]. The same type of difference was reported at slightly different wavenumbers (1000 cm^−1^ and 674 cm^−1^) in pancreatic and prostate cancer [55]. In the same sense, Saatkamp et al. [37] in a Raman spectroscopy study of urine reported estimated lower concentrations of urea and creatinine in two groups of diabetes and hypertension patients than the concentrations in healthy donors, suggesting that the differences in the concentrations of urea and creatinine in healthy donors and patients with diabetes and hypertension can be related to the severity of the complications that could lead to a decrease in renal function and culminate with a renal failure. Moreover, it was suggested that these differences can be identified using a quantitative Raman model applied to single-collection midstream urine, these results being in good agreement with another similar study [48]. Within this context, our results are supported by other reported data showing that specific alterations in the expression of the urea cycle (the main pathway by which mammals dispose of waste nitrogen) occur in many tumors, leading to a general metabolic hallmark termed “urea cycle dysregulation” (UCD) [64]. UCD elicits nitrogen diversion toward carbamoyl-phosphate synthetase 2, aspartate transcarbamylase, and dihydrooratase (CAD) activation and enhances pyrimidine synthesis, resulting in detectable changes in nitrogen metabolites in both patient tumors and their biofluids [64]. The accompanying excess of pyrimidine versus purine nucleotides results in a genomic signature consisting of transversion mutations at the DNA, RNA, and protein levels, demonstrating that UCD is a common feature of tumors that profoundly affects carcinogenesis, mutagenesis, and immunotherapy response. It is also worth mentioning that our results related to the increased intensities in the RCC cases of the SERS peaks recorded at 1156 cm^−1^ and 1296 cm^−1^ assigned in the literature to cytosine (Table 1) support the above findings.

Concerning the other SERS peaks identified in our study’s urine samples, we would like to emphasize that the lack of comprehensive databases containing well-characterized SERS spectra of urine components makes it challenging to identify and assign specific vibrational bands. The absence of standardized reference data hinders the comparison and validation of results across studies. Interdisciplinary efforts are essential for advancing the field and achieving consensus in SERS vibrational band assignments.

Given the intricate nature of biological samples and the remarkably low concentrations of analytes, establishing a sensitive and reliable method solely based on SERS peak intensity for individual sample separation remains unfeasible. To address this challenge, a multivariate statistical analysis was undertaken to enhance the discrimination of each urine specimen.

### 2.3. Multivariate Analysis

#### 2.3.1. Multivariate Analysis of Raw Data

Initially, we conducted an unsupervised Principal Component Analysis (PCA) in the first step to diminish the data dimensionality. Each spectrum originally consisted of 1015 datapoints within the wavenumber range of 353–2533 cm^−1^. We restricted this spectral range to 353–1750 cm^−1^, excluding features above 1750 cm^−1^. In the spectral range of 1750–2500 cm^−1^, we recorded only a weak peak at ~2100 cm^−1^ which we tentatively assigned to the CN triple bond stretching within the thiocyanate group (SCN). At this point, it is worth mentioning that the thiocyanate ^−^S-C≡N has a constitutional isomerism with the isothiocyanate S=C=N^−^ with different vibration characteristics. The thiocyanate is characterized by the triple bond stretching at ~2200 cm^−1^, while the isothiocyanate is characterized by the S=C=N asymmetric stretching at 2000 cm^−1^ [65,66] with the latter isomer being considered more stable. The interest in thiocyanate in biomedicine is related to its presence in human biofluids (blood, saliva, and urine) being taken from food (*Brassica* vegetables, milk, and cheese). Higher levels of thiocyanate were found in smokers from the metabolization of CN^−^ present in tobacco and it can be readily detected by SERS [67], with the strongest characteristic peak recorded at 2108 cm^−1^. Some of us have recently demonstrated that thiocyanate has the strongest peak in the SERS on a solid silver nanoparticle substrate of human saliva at 2108 cm^−1^ and its intensity increases following medical irradiation [68]. The thiocyanate level changes might be significant in kidney diseases as it was observed that individuals with elevated levels of serum SCN experience a higher risk of atherosclerosis, which further leads to some cerebral and kidney diseases [69]. However, in our case, the peak around 2100 cm^−1^ is very weak and for this reason, we chose not to include the spectral range of 1750–2500 cm^−1^ in our analysis [68]. Consequently, each spectrum was condensed to 604 datapoints. In summary, the datasets consist of 94 samples and 604 variables. PCA serves as a valuable tool for exploratory data analysis by reducing the dimensionality of extensive datasets, offering a visual representation of sample relationships, and identifying influential variables. It facilitates the unveiling of masked structures and patterns within the data, providing insights into the underlying mechanisms or factors that contribute to similarities or differences among samples.

We decided to reduce the dimensionality to 15 principal components (PCs), encompassing over 99% of the data variance. The explained variance for the 15 PCs is presented in Figure S8 and Table S1. The loading plots are depicted in Figure S9 for all 15 PCs, while detailed loading curves for the first three PCs with the vibration band contributing to the model can be found in Figure S10. To validate the model, we used the leave-one-out cross-validation (LOOCV) method. This approach involves systematically selecting one sample as the validation set while using the remaining samples for training. This process is repeated for each sample in the dataset, ensuring that every datapoint serves as a validation set at least once. The model is then assessed based on its performance across all iterations, providing a thorough validation by comparing each sample with all the others. LOOCV is particularly useful for maximizing the use of available data and obtaining a robust evaluation of the model’s generalization capability.

The first principal component explains almost 60% of the variability in the data, the second PC another 20%, while PC3 and PC4 describe 7% and 6% of data variance, respectively.

The loading plot of the first PC (Figure S10) presents positive peaks at the wavenumbers corresponding to the peaks in the SERS spectrum of urine associated with urea (1004 cm^−1^, 527 cm^−1^), and creatinine (671 cm^−1^), as well as other intense peaks at 802 cm^−1^, 892 cm^−1^ (which have a highly positive correlation with the peak at 671 cm^−1^), and at 1297 cm^−1^. The PC2 loading curve is dominated by negative peaks in the range of 1200–1400 cm^−1^ and at 393 cm^−1^. The PC3 loading plot exhibits negative peaks at 665 cm^−1^ and 889 cm^−1^ (in opposition with PC1 at almost the same wavenumbers) and a strong positive peak at 1003 cm^−1^. 

**Figure 4:**
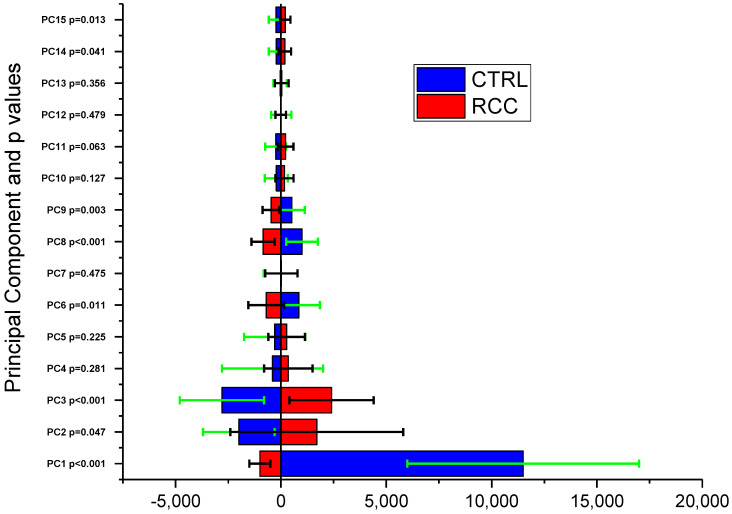
Student’s *t*-test for the scores of principal components obtained in the PCA between the RCC cases and healthy controls (CTRLs). Pearson’s coefficient *p*, the mean scores, and the standard deviations are shown. The scores are represented on the abscissa (mean +/− standard deviation). Along the vertical axes, the PC number and Pearson’s coefficient are indicated.

**Figure 5:**
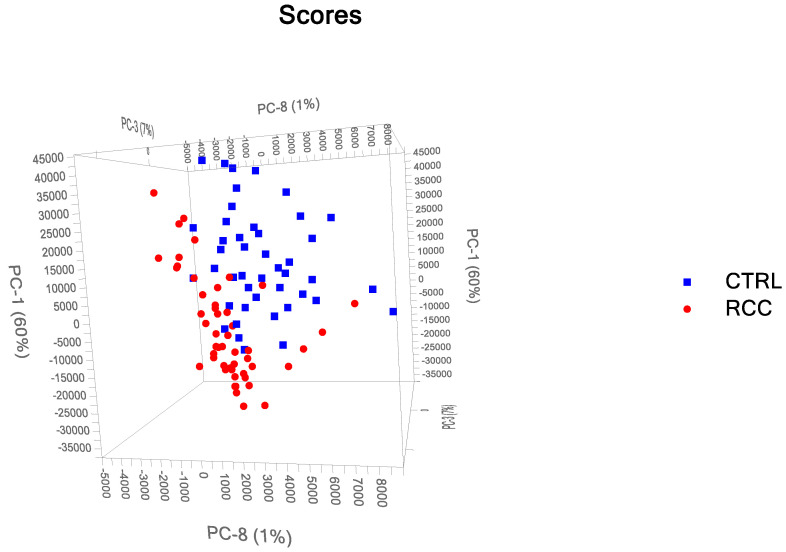
Three-dimensional (3D) scattered representation of the scores for PC1, PC3, and PC8 showing highly significant differences (*p* < 0.001) between the controls and RCC patient samples.

As expected, the loading plots are mainly related to the main SERS peaks measured in the urine samples. However, not all the PCs contribute to the discrimination between the controls and the RCC patients in a manner related to their contribution to the explained variance of the data. In this sense, we performed the Student’s *t*-test on all 15 PCs based on their scores aiming at identifying the PCs with scores significantly different between the two groups. The *p*-coefficients and the means +/− standard deviations are presented in Figure 4. Highly significant differences between the two groups (*p* < 0.001) were obtained for PC1, PC3, and PC8 and significant differences (0.010 < *p* < 0.05) for PC2, PC6, PC9, PC14, and PC15 (Figure 4). In Figure 5, we used a 3D representation of the scores using the PCs with highly significant differences between the two groups. 

**Figure 6:**
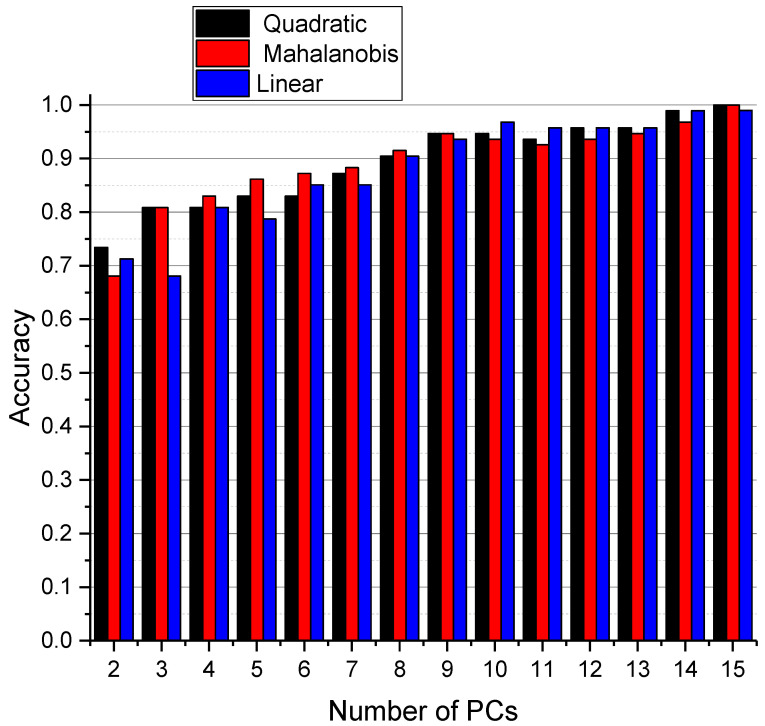
Accuracy of discrimination between controls and RCC cases with different numbers of PCs and three discrimination functions: quadratic (black), Mahalanobis (red), and linear (blue).

**Figure 7:**
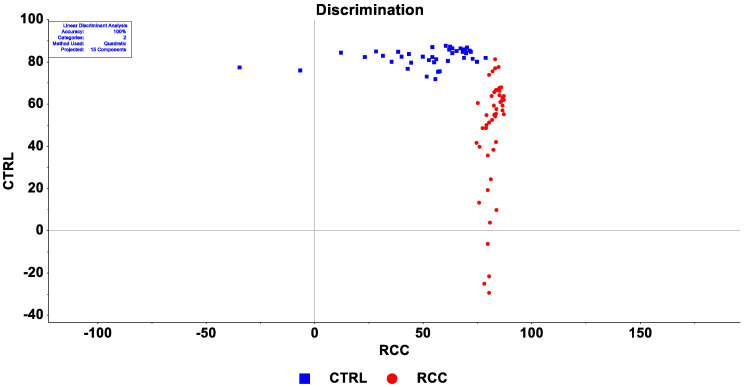
Discrimination plot between the RCC and CTRL cases obtained using the PCA-LDA with 15 PCs and the quadratic discrimination function.

One can notice that, even in the case of an unsupervised analysis, like the PCA, the sample points of the two groups are relatively well separated. However, it is obvious that only three PCs do not fully discriminate the two groups and more components are needed in this respect. In the subsequent phase, we systematically applied a supervised classification method to distinguish between the two datasets. We employed linear discrimination analysis combined with Principal Component Analysis (LDA-PCA). The outcomes were achieved by considering up to 15 principal components (PCs) and utilizing three discrimination functions, namely, the linear, quadratic, and Mahalanobis. To assess the model’s performance, we employed the same leave-one-out cross-validation (LOOCV) approach as before, but this time within the context of a supervised model. In this scenario, each sample was compared with all the others, taking into account the category to which the other samples belonged. With the specified parameters, the discrimination accuracy reached 100% for all three functions. The accuracy of discrimination for the three discrimination functions and different numbers of PCs considered is presented in Figure 6, while the sensitivity and specificity are shown in Figures S11 and S12. Also, in Table S2, we provide all the data in detail and we define the accuracy, specificity, and sensitivity. The discrimination plot for 15 PCs is presented in Figure 7 for the quadratic discrimination function and Figures S13 and S14 for the Mahalanobis and linear discrimination functions, respectively. As expected, the figures of merit increase as the number of PCs taken into account increases, regardless of the discrimination function used in the analysis. Also, for up to nine PCs considered, the non-linear (quadratic and Mahalanobis) functions perform better as compared to the linear function. Above eight PCs, the accuracies of determination are higher than 0.9 for all discrimination functions considered. The figures of merit reported here on urine are slightly lower as compared to those obtained in the discrimination of RCC based on label-free SERS of blood serum obtained in a previous study by our group [32]. In that study, we obtained a 100% accuracy for 12 PCs using the quadratic discrimination function. These small differences might be explained by the fact that the serum is richer in molecules with higher affinity for the SERS substrate which have different concentrations between the RCC patients and the control group (e.g., uric acid). However, our data are significantly higher than the maximum 0.8 classification accuracy obtained by another group using three classification algorithms (random forest, kNN, and naïve Bayes) [70]. These results demonstrate that the free-label SERS analysis of urine is a powerful tool for discriminating the RCC cases from healthy controls. 

We also tried to test our approach for the discrimination of the RCC patients according to the stage of the disease. Since in the LDA-PCA algorithm, the number of components equals the minimum number of samples in a class, we used seven PCs as out of 50 RCC cases, 32 were diagnosed in stage 1, 11 in stage 3, and 7 in stage 3. The confusion matrix of the analysis performed on raw data using seven components and a quadratic discrimination function are presented in Table S3. From the 94 samples, 78 were correctly assigned to their class (40 out of 44 for CTRL, 27 out of 32 for stage 1, 7 out of 11 for stage 2, and 4 out of 7 for stage 3) yielding an overall accuracy of 83%.

Another method to increase the accuracy of discrimination is to select the principal components that present significant distinct values between the controls and RCC cases (see Figure 4). In this sense, using the first three PCs with the highest significant difference between the two classes, we obtain an overall accuracy of 85% for all three discrimination functions, which is noteworthily increased as compared to the accuracy obtained by using the first three PCs on both raw and normalized spectral data. Moreover, using the six components with the largest differences between the two classes, we obtained an accuracy of 95% in discriminating the controls from RCC cases and of 97% with seven components.

We applied the same strategy for discriminating the samples according to the stage. We obtained this by using seven components (PC1, PC2, PC3, PC6, PC8, PC14, PC15) with an overall accuracy of 88%, (83 samples out of 94 being correctly assigned to their classes), which is significantly improved as compared to the results obtained using the first seven PCs (Table 2).

#### 2.3.2. Multivariate Analysis of Normalized Data

The data presented until this point were obtained using the raw SERS data, with the measurements being performed under the same conditions for all samples. We also performed statistical and multivariate analyses after the data were normalized. We used the area-normalization method as in the case of our previous study [32] where area normalization was proved to yield the highest discrimination accuracy between the controls and RCC patient cases. The mean SERS spectra for both RCC and CTRL cases and the difference spectrum are presented in Figure S15. Once again, the difference spectrum shows a higher SERS peak intensity for CTRL cases at 1004 cm^−1^ which was assigned to urea. 

We also studied the correlations between the variables (Figure S16) for area-normalized SERS spectra. As compared to the case of raw data, we noticed a less reduced degree of correlation between the variables (Raman shifts), with high coefficients of correlation (>0.9) being calculated only in the spectral range of 1200–1450 cm^−1^. The loading plots for the first three PCs are presented in Figure S17. For PC1, we noticed in the loading plot a major positive peak at 1004 cm^−1^ and other positive peaks at 670 cm^−1^ and 889 cm^−1^, being very similar from this point of view with the loading plot of PC1 in the case of raw data. However, in the case of area normalization, there are three negative peaks at 1235 cm^−1^, 1331 cm^−1^_,_ and 1405 cm^−1^ for PC1, which are also calculated for the PC2 loading plot in the case of raw data. This observation suggests that after area normalization of the raw SERS data, the PCs contain more information. 

**Figure 8:**
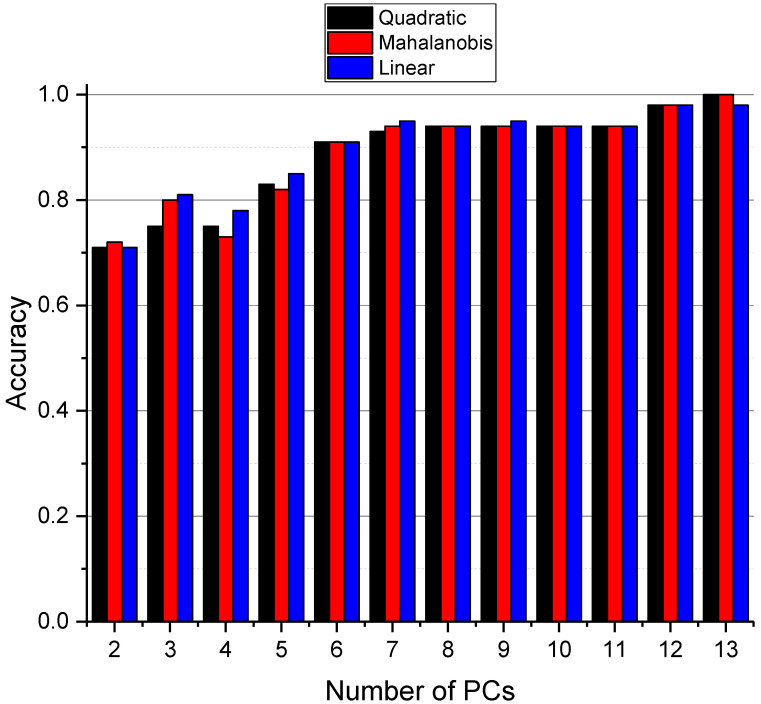
Accuracy of discrimination between RCC cases and control healthy donors as a function of the number of PCs, using LDA-PCA for area-normalized data and the quadratic (black), Mahalanobis (red), and linear (blue) discrimination functions.

We checked the effect of the data normalization on the accuracy of discrimination between the controls and RCC patients’ cases. The analysis disclosed an increase in the accuracy of determination with 100% accuracy reached with 13 PCs using either the quadratic or Mahalanobis discrimination functions (Figure 8) for the area-normalized data. Moreover, accuracy values higher than 91% are obtained for the analysis taking into account only six PCs with all three discrimination functions (Figure 8), all these results show that the spectral normalization improves significantly the discrimination results. We also notice that the accuracy of discrimination increases with the number of principal components considered. This increase is steeper up two six PCs, with a higher number of PCs the accuracy values reach a plateau or increase only very slightly. 

**Figure 9:**
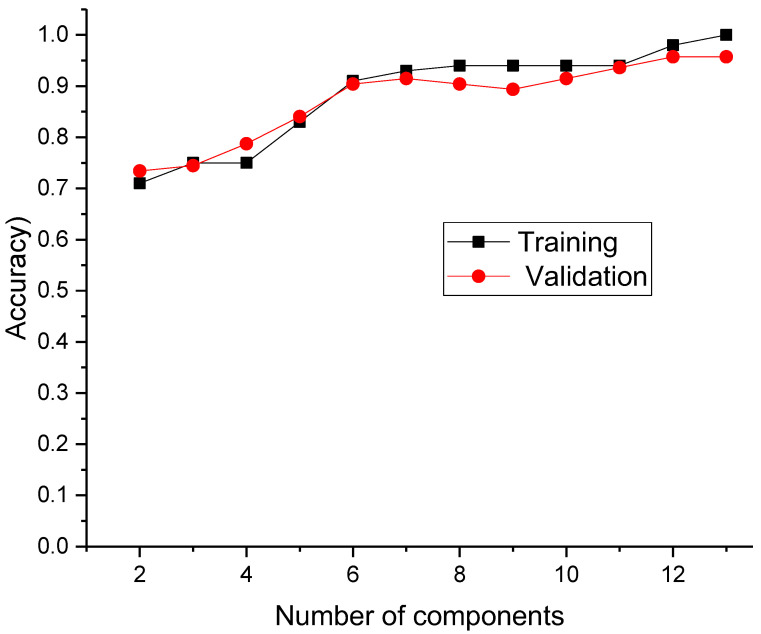
Accuracy of discrimination between the RCC and CTRL cases for the training set (based on map 1 data) and the validation set (on map 2 data) using LDA-PCA with a variable number of PCs (2–13) and a quadratic discrimination function.

Aiming at checking the performance of the built models on the new data, we considered splitting the data in two sets used for training and validation, respectively, with the main goal to compare the discrimination accuracy features on these two sets [71,72]. In this sense, the data were separated in two sets, denoted map 1 and map 2. As mentioned previously, for each sample we recorded 100 datapoints delimitated in two different maps with 50 datapoints on each map. By splitting the data in this manner, we avoid having two sets of data from the same sample in the same class, and we have 94 samples both in the training and in the validation sets. Based on the data recorded on map 1, we built several LDA-PCA models with an increased number of components between 2 and 13. For all the models, we estimated the accuracy figures on the training set. There are very minor differences for the discrimination accuracies calculated in this manner as compared to the accuracies calculated for both maps (100 datapoints). Afterward, all these models created based on map 1 were applied to the data recorded in map 2 for prediction. In this manner, we calculated the accuracy for the validation. The results are presented in Figure 9. 

As can be seen from Figure 9, although the discrimination accuracies are slightly lower for the validation as compared to the training set, their values are very similar. Once again, we see a steep increase in the accuracy of discrimination up to six PCs, followed by a plateau. Based on this observation, we can conclude that the tradeoff between the accuracy and the number of components corresponds to about six PCs. However, based on the explained variance data (Figure S8, the Scree Plot) and the elbow rule, we notice that up to four PCs the explained variance increases steeply (corresponding to about 90% of the explained variance), followed by a much smaller slope above four PCs. The latter result would indicate an optimal number of four PCs. 

However, in PCA, which is an unsupervised algorithm, the variance is related to both intraclass and between class variances. Therefore, in the following, we studied the influence of how different PCs taken into account for the LDA, influence the discrimination accuracy, showing that choosing the PCs with the highest difference between classes can reduce the number of PCs for obtaining similar discrimination accuracy figures. In this sense, we applied the Student’s *t*-test of the scores calculated from the PCA for the first 13 PCs (Table S4). The highest significant differences were calculated for PC1 and PC5 (*p* < 0.001) followed by PC7 and PC8 (*p* = 0.002). Applying the LDA for two principal components, PC1 and PC5, led to a discrimination accuracy of 80–81% and 88% for the three principal components (PC1, PC5, PC7), which are significantly higher as compared to the case in which we take into account the first PCs according to their explained variance values (Table 3).

It is quite obvious that choosing the PCs with the highest significant differences between the two classes leads to a significant increase in the accuracy of discrimination, the most remarkable increase being calculated for the linear discrimination function. In the end, we obtained the accuracy of the discrimination of 90% for three PCs and a linear discrimination function, mitigating practically the risk of overfitting and increasing the robustness of our analysis. 

### 2.4. Supported Vector Machine (SVM) Analysis

Recently, it has been suggested that Supported Vector Machine (SVM) classification may offer superior discrimination in LF-SERS of RCC compared to LDA-PCA [73]. SVM, a widely used pattern recognition technique in data mining applications, was applied to our data using the Unscrambler^®^ software, based on the code developed by Chang and Lin [74]. The kernel trick in Support Vector Machines (SVMs) is a computational technique that allows SVMs to efficiently handle non-linearly separable data by implicitly mapping the data into a higher-dimensional space without actually transforming the data. In SVM, the linear kernel, being the simplest, is less prone to overfitting, and thus, it was selected for our data analysis.

For C-type SVM, a capacity factor/penalty constant C needed to be set. We optimized the selection of C using a grid method, varying C to optimize accuracy. A coarse grid search was initially performed, followed by a more detailed “fine” grid search. The cross-validation method used was a 94-segment cross-validation, which is equivalent to an LOOCV. Sequentially, one sample was tested using the classifier trained on the remaining 93 subsets, thus preventing overfitting. The SVM algorithm with a linear kernel function applied to the raw data achieved a classification accuracy of 100% for the training set and 98.9% for the validation set, with 22 support vectors.

Similar discrimination accuracy was obtained for area-normalized data, with 100% accuracy for the training set and 98.93% for the validation set. Although the SVM method does not restrict the number of samples in a set, when applied to classifying samples based on the stage, the results (Table S5) revealed an overall accuracy of 81%. All control samples (44) and stage 1 samples (32) were correctly classified. However, all 11 samples of stage 2 were classified as stage 1, and all 7 samples of stage 3 were classified as stage 1. It seems that the SVM classifier, at least in the form implemented in Unscrambler X 10.5.1 software is limited to a binary classification, and therefore in the case of several classes, more complex algorithms should be used.

Our study reveals that Principal Component Analysis-Linear Discriminant Analysis (PCA-LDA) outperforms Support Vector Machine (SVM) in providing discrimination scores, especially in showcasing meaningful differences despite their seemingly minimal nature. Beyond superior discrimination scores, PCA-LDA offers insights into the most significant contributing components, shedding light on the metabolites driving the observed distinctions. This nuanced information is crucial for comprehending the biochemical basis of group differences and facilitating more insightful interpretations.

SVM, while enabling the identification of supporting vectors, presents challenges in correlating them with SERS spectra. The process of linking supporting vectors with SERS spectra is more intricate and less intuitive compared to the clear connection established by PCA-LDA. From an interpretability standpoint, PCA-LDA emerges as more advantageous for this study. Overall, our data emphasize that label-free SERS, coupled with chemometrics, enable the identification and analysis of specific molecular signatures associated with cancer, particularly renal cell carcinoma (RCC).

Chemometrics plays a pivotal role in processing and interpreting the vast amount of spectral data generated by label-free SERS, extracting relevant information from complex spectra, and enhancing the accuracy and reliability of cancer detection. The integration of label-free SERS with chemometrics offers advantages such as eliminating the need for exogenous labels or dyes, simplifying detection, and reducing potential interference. Moreover, it allows for high sensitivity, specificity, and multiplexing capabilities, facilitating the simultaneous detection of multiple cancer biomarkers.

One limitation of our study is the use of a state-of-the-art Raman spectrometer, and for true clinical translation, considerations for a low-cost, point-of-care device are essential. One important result of our study is that urine could be explored for its potential in disease diagnosis, especially in kidney cancer, with our data on urine being similar to those obtained by us in a previous study on blood serum [32] and outperforms the results of other studies made in blood components [70].

The adoption of label-free Surface-Enhanced Raman Scattering (SERS) of urine for cancer differentiation faces challenges in routine clinical practice due to the need for more data to distinguish between various cancer types. Establishing distinctive SERS signatures for different cancer types is crucial, considering the release of different biomarkers into the urine. However, the lack of a comprehensive library of cancer-specific SERS spectra poses a challenge in reliable distinction between cancer types. Biological variability among patients adds complexity to distinguishing cancer types based on SERS data. While label-free SERS of urine holds promise, further extensive data collection and research are needed to fully exploit its potential for distinguishing between different types of cancer in routine clinical practice. 

## 3. Materials and Methods

Silver nitrate (AgNO_3_) and hydroxylamine (NH_2_OH–HCl) from Roth GmbH (Gladbach, Germany) were of analytical grade and used as received. CaF_2_ polished glasses (Crystran Limited, Poole, UK), featuring a single Raman peak at 321 cm^−1^, served as solid substrates.

### 3.1. Nanoparticle Synthesis and Preparation of the Substrate

The solid plasmonic substrates were crafted using an innovative method developed by our team at the MEDFUTURE Research Center for Advanced Medicine, University of Medicine and Pharmacy Cluj-Napoca [31]. Silver nanoparticles (AgNPs) were synthesized by reducing silver nitrate with hydroxylamine, following the procedure proposed by Leopold and Lendl [75]. Briefly, a solution comprising 12 mg of NaOH and 10.4 mg of NH_2_OH HCl dissolved in 90 mL of de-ionized water was stirred (500 rpm). To this solution, 10 mL of another solution containing 17 mg of AgNO_3_ was added. The mixture underwent an immediate color change to brown, then yellowish. The resulting colloid exhibited an absorption spectrum with a peak around 408 nm. The silver colloids were purified and concentrated to 10× using tangential flow filtration (TFF, Pall Corporation, New York, NY, USA) [31,76]. This filtration step enhanced the SERS performance by reducing polydispersity and eliminating synthesis byproducts. The CaF_2_ glass was cleaned with acetone and ethanol, rinsed with ultrapure water, and air-dried. After 15 min, the port probe was heated to 40 °C using a plate heater. Subsequently, 1 microliter of concentrated colloid was applied to the CaF_2_ port probe and allowed to dry for 80 s. Once prepared, the SERS solid substrates were cooled to room temperature and ready for use, consistently delivering reproducible SERS spectra of biological fluids [31,76].

### 3.2. Analyte Deposition and Surface-Enhanced Raman Spectroscopy (SERS) Measurements

A 1 μL volume of urine was pipetted onto the dry solid substrates, left to dry at room temperature, and subjected to SERS measurements. Spectra were acquired using a Renishaw inVia Reflex Raman confocal multilaser spectrometer (Renishaw plc, Gloucestershire, UK) with a resolution of approximately 2 cm^−1^, utilizing a laser excitation wavelength of 785 nm. A 50× objective lens (N.A. = 0.75) of a Leica microscope directed the laser beam to the sample. Spectra were recorded using a 600 lines/mm grating and a charge-coupled device (CCD) camera, with a laser power set at 1% of the maximum power, the power at the sample being 2.2 mW. The laser beam was focused approximately 40–50 μm from the dried sample edge, and data were collected in mapping mode, with an acquisition time of 10 s for each of the 50 spectra from different points in two maps for each sample. Wire 4.2 software provided by Renishaw in conjunction with the inVia spectrometer was employed for baseline correction and cosmic rays removal.

### 3.3. Research Ethics

All participants provided informed written consent according to the Declaration of Helsinki 2013. The study protocol was approved by Cluj-Napoca Municipal Clinical Hospital, Ethics Committee Decision No. 1 from 19 January 2018, for the study entitled “Biomarkers for early diagnosis of bladder, prostate, and kidney cancer by Raman spectrophotometric profile analysis of biological fluids (blood and urine) and humoral tissues involving humans”.

### 3.4. Cohort of Patient Samples

A total of 118 patient samples were collected from individuals diagnosed with renal tumors, following contrast-enhanced computed tomography (CECT), with each individual providing informed written consent. The research included 50 male patients with RCC and 44 healthy male donors. Samples from RCC patients were gathered prior to undergoing surgery or receiving any form of therapy. The diagnosis of these patients was validated post-surgery through histopathological examination. To decrease sample variability, the study was restricted to male participants. Moreover, among cancer patients, only those with a diagnosis of clear-cell RCC were considered for inclusion. Patient ages ranged between 38 and 78 years for RCC patients and from 19 to 88 years for healthy donors. Demographic, type of cancer, and statistical data on the cohort of participants are provided in Tables S6–S8.

### 3.5. Urine Collection

Urine samples were obtained from participants after overnight fasting in sterile recipients and stored at −80 °C until SERS analysis.

### 3.6. Multivariate Analysis

Multivariate analysis of SERS spectra was performed using Unscrambler X 10.5.1 software (Camo Analytics, Oslo, Norway). OriginPro 2016 software (OriginLab, Northampton, MA, USA) was used for graphical representation and some simple statistics. In the case of PCA, we employed the leave-one-out cross-validation (LOOCV) technique to validate the model. This method entails systematically designating one sample as the validation set, utilizing the remaining samples for training. This process iterates for each sample in the dataset, guaranteeing that every datapoint acts as a validation set at least once. The model’s performance across all iterations is then evaluated, offering comprehensive validation by comparing each sample against all others. LOOCV proves especially beneficial for optimizing data utilization and obtaining a robust assessment of the model’s ability to generalize.

A similar approach was used for the validation of the model in the case of SVM. The cross-validation method used was a 94-segment cross-validation, which is equivalent to an LOOCV. Sequentially, one sample was tested using the classifier trained on the remaining 93 subsets, thus preventing overfitting.

## 4. Conclusions

The findings presented in this study highlight the potential of label-free Surface-Enhanced Raman Spectroscopy (SERS) on human urine, coupled with multivariate and machine learning techniques, as a valuable tool for distinguishing renal cell carcinoma (RCC) cases from healthy donors. Both LDA-PCA and SVM algorithms demonstrated high accuracy in discriminating between the two classes, with LDA-PCA exhibiting better discrimination among different stages of RCC (88% accuracy), compared to SVM.

We conducted a statistical analysis of the raw spectral data to identify the key vibrational bands responsible for discriminating between the two classes. Our results indicate that SERS peaks associated with urea and creatinine, the major urine components, significantly contribute to discrimination. However, additional SERS peaks in urine samples, not definitively attributed to blood components, play an essential role in the discrimination process. Urine biomarkers play a pivotal role in detecting cancer presence, recurrence, and progression, offering real-time insights that can be monitored through SERS. Nonetheless, further studies are required for the accurate assignment of urine SERS peaks and increasing the relevance of SERS of urine in cancer screening.

In the case of LDA-PCA, we analyzed the impact of preprocessing steps on discrimination. While raw SERS data could reveal relative concentration differences in urine metabolites, normalization of spectral data substantially enhances discrimination accuracy in cancer cases. Additionally, selecting principal components with significantly different scores between the two classes reduces the risk of overfitting and minimizes the number of components used for discrimination.

Label-free SERS of urine shows promising potential for RCC screening. However, before clinical translation, more clinical cases and comparisons with other types of cancers and organ-specific diseases are essential for a comprehensive understanding of its diagnostic capabilities.

## Figures and Tables

**Table 1:** Main vibration peaks for the SERS of urine samples and their assignments using laser excitation lines between 633 and 830 nm.

Raman Shift Measured [cm^−1^]	Raman Shift Range Reported [cm^−1^]	Assignments in Literature	Tentative Assignments in This Study
525	495–533	Cholesterol ester/ nucleic acids [33,34,35,36] Creatinine [37] Urea [38,39] Albumin [40,41] Uric acid [36]	Urea
596	575–605	Albumin [42] Creatinine [39,43,44] Urea [39]	Creatinine
671	635–702	Uric acid [33,34,35] Creatinine [38,39,44,45,46,47,48] Urea [37] Cholesterol [49] Xanthine [44] Collagen type I [40] DNA and proteins [36,41] Tyrosine [36]	Creatinine
721	725–744	Adenine, coenzyme A [33,36] Creatinine [38] Phosphatidylserine [40] Uric acid [34,35] Hypoxanthine [34,35] DNA [36]	Hypoxanthine/Adenine
804	797–813	Albumin [33,34,35] Creatinine [38] Phosphodiester [34,49] DNA [41] Uric acid [36] Serine [36]	Uric acid/Creatinine
893	839–912	D-Galactosamine [33,34,35,36] Creatinine [38,39,47,48,50] Urea [37] Albumin [42,49] Calcium oxalate [40] Hydroxybutyrate [39] Acetoacetate [39] Nitrogenous compounds [39] Isoprostanes [39]	Uric acid/Creatinine
941	958–959	Urea [38] Creatinine [50] Hydroxyproline [34]	?
1004	999–1096	Phenylalanine [33,41] Urea [34,35,36,37,38,39,42,44,45,46,47,49,50,51] Creatinine [37,52] Albumin [42,49,53,54] Hydroxybutyrate [39,52] C–C aromatic ring stretching [40] Phosphate [39]	Urea
1156	1128–1173	Phenylalanine [33,34,35,36] Creatinine [37] Glucose [48] Urea [38,39] Cytosine/guanine [49] C–C skeletal stretching [40] Albumin [41] Tyrosine [41] D-mannose [36]	Cytosine?
1235	1206–1250	Albumin [50] Xanthine [55] Phenylalanine [34,41] Tryptophan [41]	?
1296	1285–1308	Cytosine [40,49] Glutamine [41]	Cytosine?
1335	1323–1383	Guanine [33,36,40] Creatinine [56] Albumin [50] CH_3_ band [40,49] Tryptophan [40] Adenine [34,35,36] DNA/RNA bases [34,35,36] Hydroxybutyrate [39] Nucleic acids [41]	ketone bodies?
1405	1420–1490	Tryptophan [33,34,35] Creatinine [39,43,47,50] Albumin [38,42] Acetoacetate [39] Hydroxybutyrate [39] Glutamine [41]	ketone bodies?
1598	1579–1615	C=N and C=C stretching [40] Phenylalanine [34,35,41] Albumin [57] Urea [39] Tyrosine [41] Phenylalanine [41]	C=N and C=C stretching
1675	1621–1745	Tryptophan (IgG) [49] Carbonyl stretch(C=O)/cholesterol ester [40] C=C stretch vibration [40] Creatinine [44] Lipids [41] Phospholipids [41]	Carbonyl stretch/C=C stretch vibration

?—for the row marked with question marks the vibrations were not assigned or the assignment is not clear.

**Table 2:** Confusion matrix for the classification of the samples based on stages using LDA-PCA and seven PCs with the highest significant difference between the classes (PC1, PC2, PC3, PC6, PC8, PC14, PC15) and a quadratic function.

	CTRL	Stage 1	Stage 2	Stage 3	Predicted
**CTRL**	43	2	0	0	**45**
**Stage 1**	1	27	1	3	**32**
**Stage 2**	0	3	10	1	**14**
**Stage 3**	0	0	0	3	**3**
**Actual**	**44**	**32**	**11**	**7**	

**Table 3:** Accuracy of discrimination between the RCC and CTRL cases in the discrimination analysis as a function of the principal components considered.

Discrimination Function	Two PCs		Three PCs	
	PC1 and PC5	PC1–PC2	PC1, PC5, PC7	PC1–PC3
Linear	81	71	90	68
Quadratic	80	73	86	80
Mahalanobis	81	68	88	81

## Data Availability

The data presented in this study are available upon reasonable request addressed to the corresponding authors. The processed data are contained within the article.

## Supplementary Materials

The following supporting information can be downloaded at: https://www.mdpi.com/article/10.3390/ijms25073891/s1.
