# Supplementary material for: Label-Free SERS of Urine Components: A Powerful Tool for Discriminating Renal Cell Carcinoma through Multivariate Analysis and Machine Learning Techniques
Source: Int J Mol Sci. 2024 Mar 31;25(7):3891. doi: 10.3390/ijms25073891 (PMC11011951; doi:10.3390/ijms25073891)
Supplement: Supplementary file 1 [file ijms-25-03891-s001.zip › ijms-2922209-supplementary.pdf]

Supplementary Materials

Label-Free SERS of Urine Components: A Powerful Tool for Discriminating Renal Cell Carcinoma through Multivariate Analysis and Machine Learning Techniques

Bogdan Adrian Buhas[1,2,3], Valentin Toma[4], Jean-Baptiste Beauval[1], Iulia Andras[2,5], Răzvan Couți[3], Lucia Ana-Maria Muntean[6], Radu-Tudor Coman[5], Teodor Andrei Maghiar[3], Rareș-Ionuț Știufiuc[4,7*], Constantin Mihai Lucaciu[7*] and Nicolae Crisan[2,5]

1 Department of Urology, La Croix du Sud Hospital, 52 Chemin de Ribaute St., 31130 Quint Fonsegrives, France; buhasbogdan@yahoo.co.uk (B.A.B.); jbbeauval@gmail.com (J.-B.B.)

2 Department of Urology, Clinical Municipal Hospital, 11 Tabacarilor St., 400139 Cluj-Napoca, Romania; dr.iuliaandras@gmail.com (I.A.); drnicolaecrisan@gmail.com (N.C.)

3 Faculty of Medicine and Pharmacy, University of Oradea, 1 Universitatii St., 410087 Oradea, Romania; razvan.couti@gmail.com (R.C.); teodormaghiar@yahoo.com (T.A.M.)

4 Department of Nanobiophysics, MedFuture Research Center for Advanced Medicine, "Iuliu Hatieganu" University of Medicine and Pharmacy, 4-6 Pasteur St., 400337 Cluj-Napoca, Romania; val-entin.toma@umfcluj.ro

5 Faculty of Medicine, "Iuliu Hatieganu" University of Medicine and Pharmacy, 8 Victor Babes St., 400347 Cluj-Napoca, Romania

6 Department of Medical Education, "Iuliu Hatieganu" University of Medicine and Pharmacy, 8 Victor Babes St., 400347 Cluj-Napoca, Romania; ana.muntean@umfcluj.ro

7 Department of Pharmaceutical Physics–Biophysics, Faculty of Pharmacy, "Iuliu Hatieganu" University of Medicine and Pharmacy, 6 Pasteur St., 400349 Cluj-Napoca, Romania

8 Nanotechnology Laboratory, TRANSCEND Research Center, Regional Institute of Oncology, 700483 Iași, Romania

* Correspondence: rares.stiufiuc@umfcluj.ro clucaciu@umfcluj.ro; Tel.: +40-744647854 (C.M.L.)

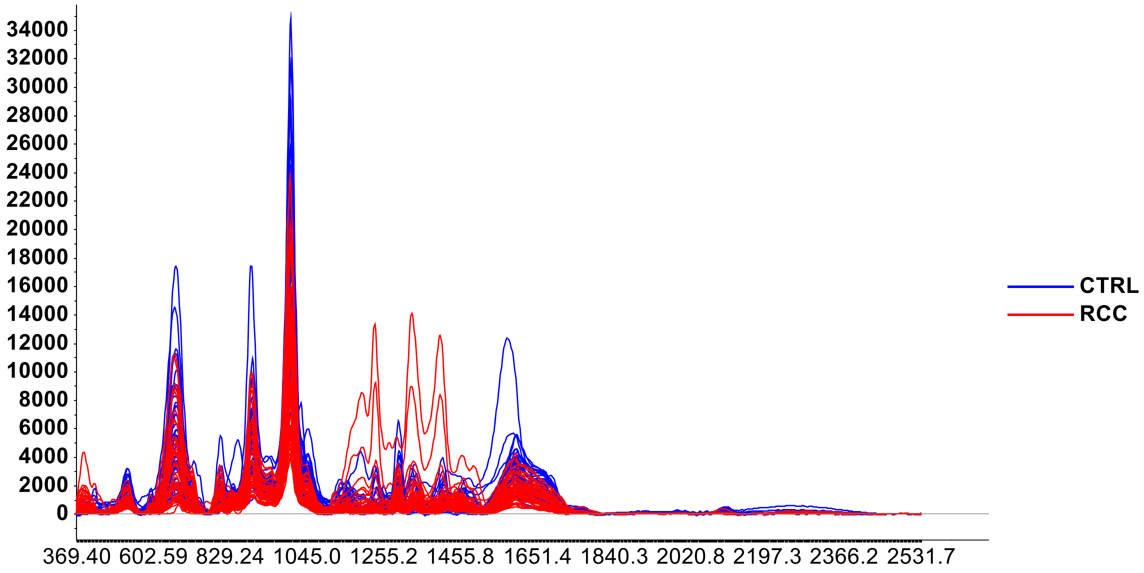

Figure S1. Raw SERS spectra of urine samples from controls (blue) and RCC patients (red)

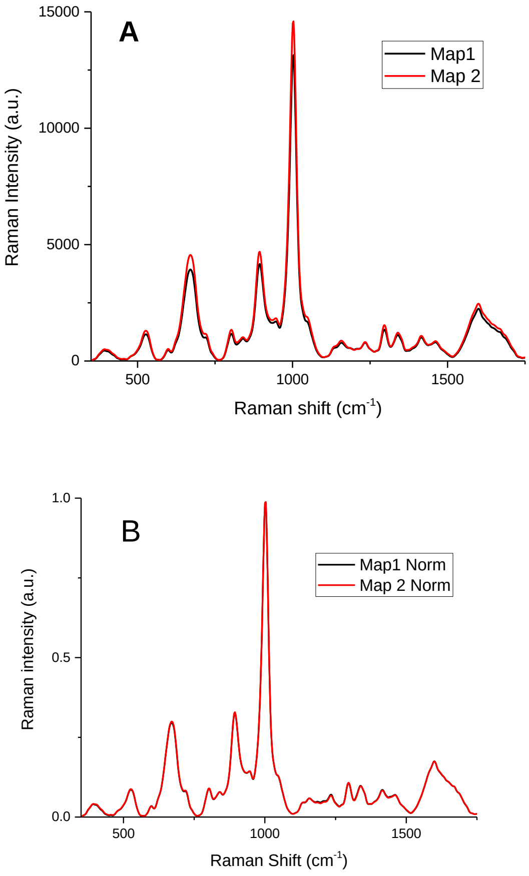

Figure S2. A)Mean SERS spectra measured for all samples on the two maps. Each map consists of 50 SERS spectra collected from different 50 spot points on the substrate. B) Superposition of the mean SERS spectra collected on the two maps after maximum normalization

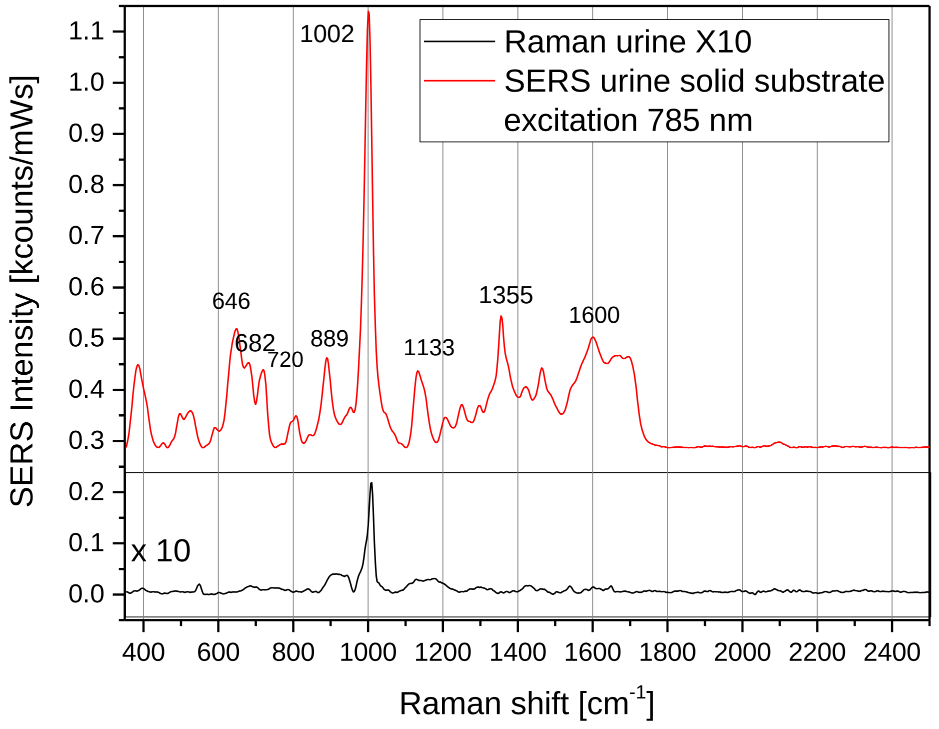

Figure S3. Raman spectrum of an urine sample (black) with a laser excitation wavelength of 785 nm and the SERS of the same sample (red) deposited on the solid substrate using he same excitation conditions. The spectra were offset for clarity. In the case of the Raman spectrum the scale is increased 10 times.

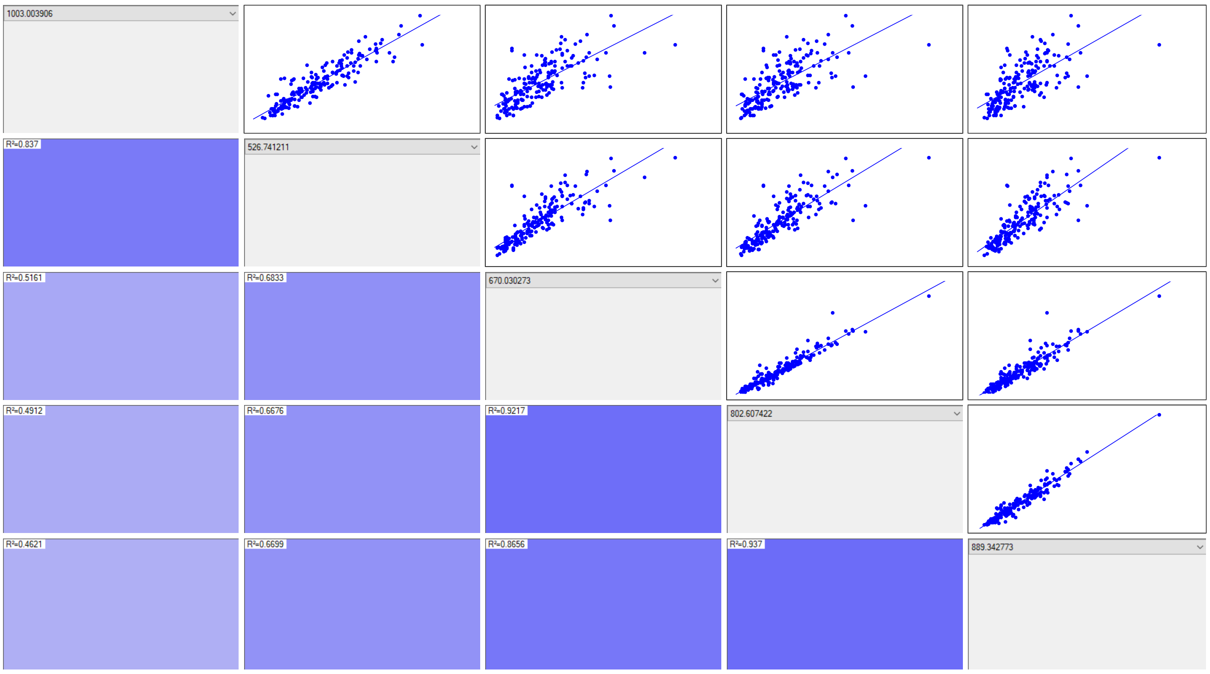

Figure S4. Correlation graphs for the vibrational bands recorded at 1003 cm^{-1}, 525 cm^{-1}, 670 cm^{-1}, 803 $cm^{-1,}$ and 889 cm^{-1}. The coefficients of determination are indicated for each pair of wavenumbers. The blue backround color intensity is proportional to the coeffieicient of determination.

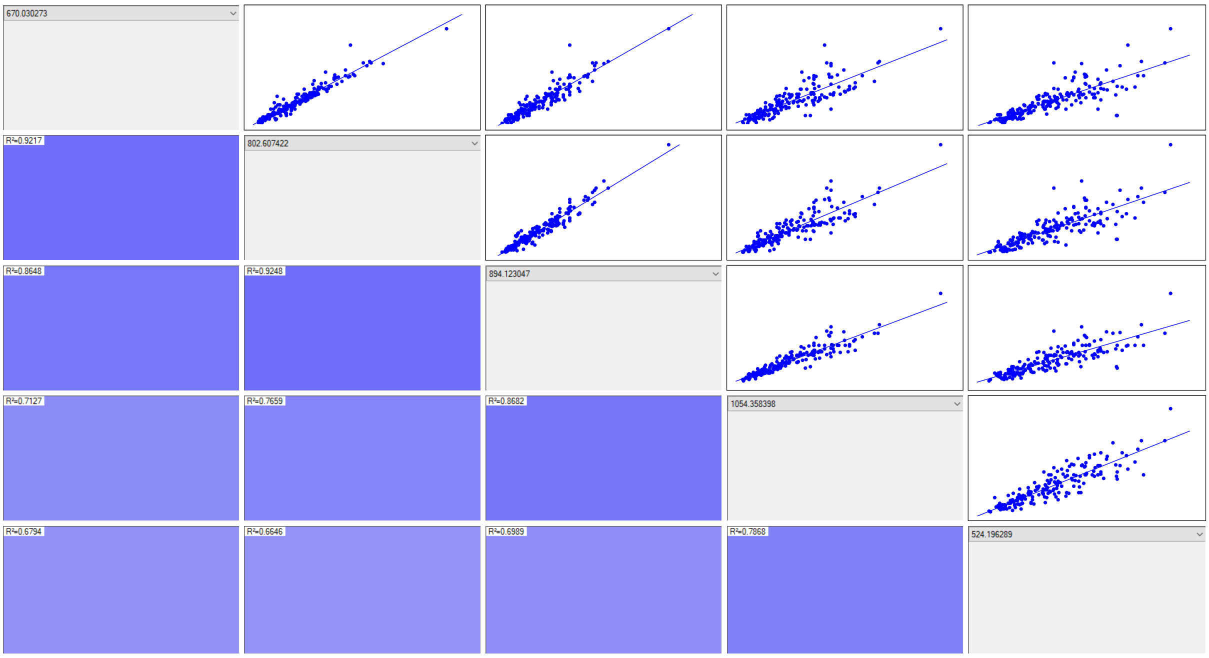

Figure S5. Correlation graphs for the vibrational bands recorded at 670 cm^{-1}, 803 cm^{-1}, 893 cm^{-1}, 1054 cm^{-1} and 524 cm^{-1}. The coefficients of determination are indicated for each pair of wavenumbers. The blue backround color intensity is proportional to the coeffieicient of determination.

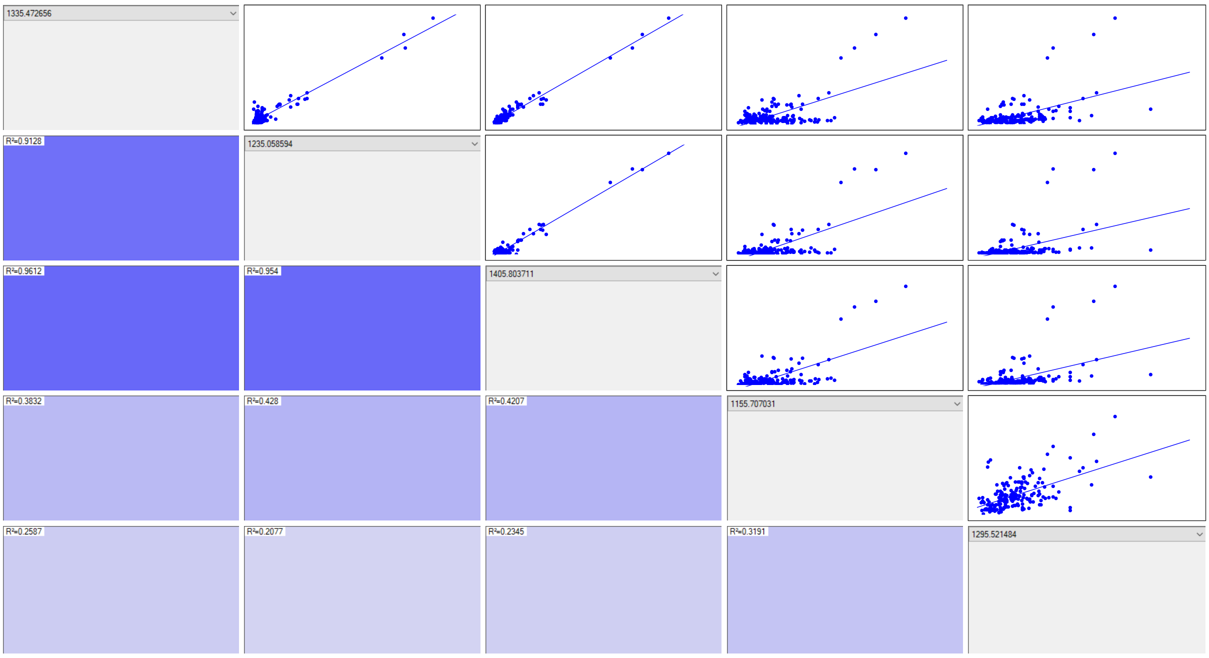

Figure S6. Correlation graphs for the vibrational bands recorded at 1335 cm^{-1}, 1235 cm^{-1}, 1405 cm^{-1}, 1154 cm$^{-1,}$ and 1296 cm^{-1}. The coefficients of determination are indicated for each pair of wavenumbers. The blue backround color intensity is proportional to the coeffieicient of determination.

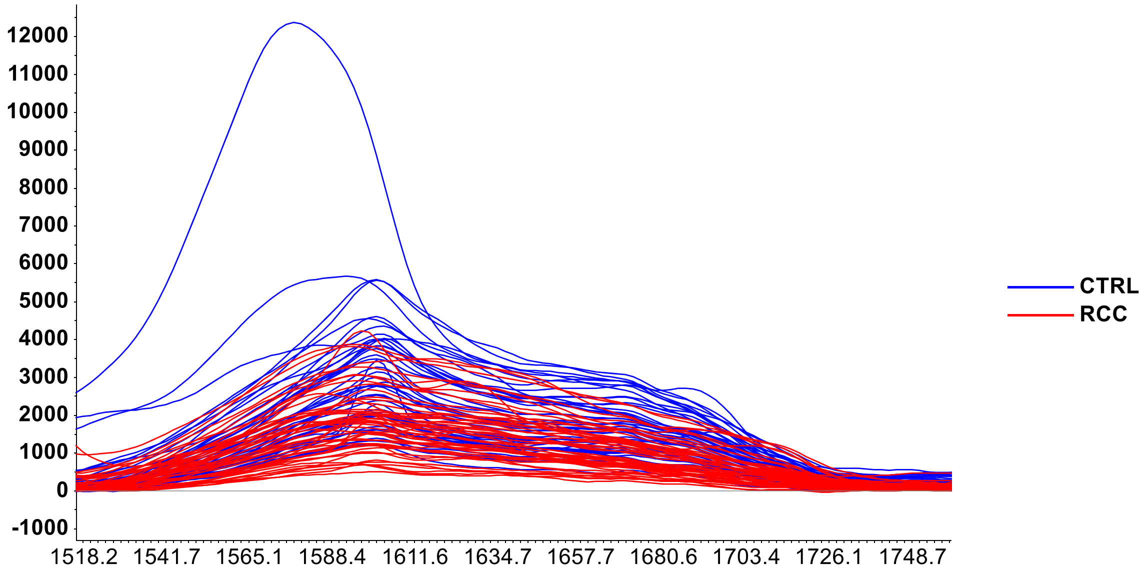

Figure S7. SERS spectra of urine for CTRL (blue) and RCC patients (red) in the wavenumbers range 1475-1750 cm^{-1}.

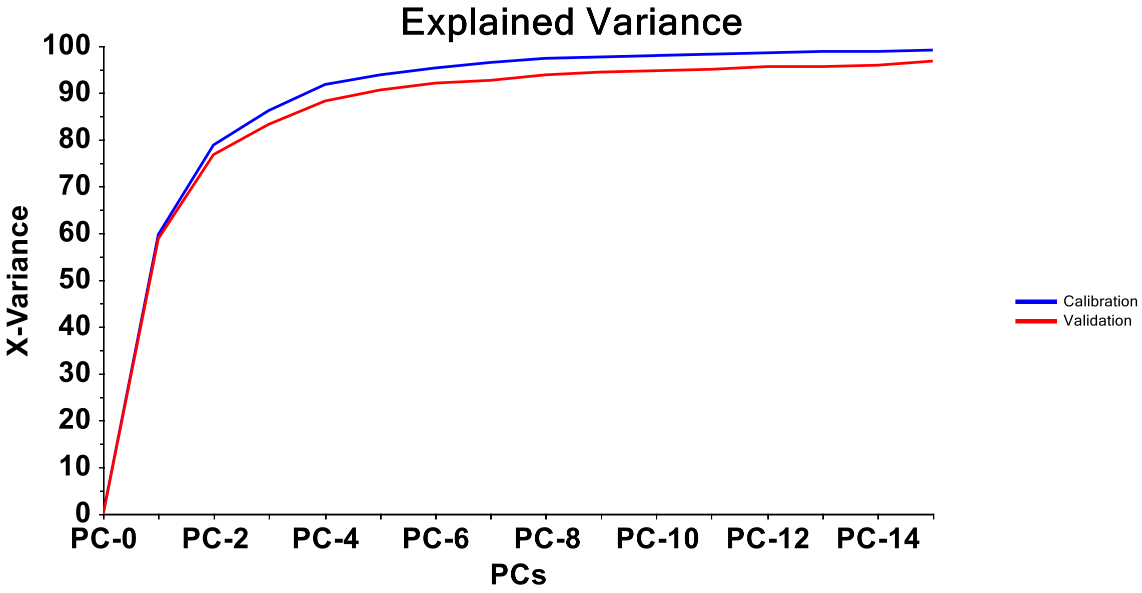

Figure S8. Explained variance for the PCA using 15 principal components.

Table S1. Explained variance for 15 PCs in the PCA

Explained Variance	PC-1	PC-2	PC-3	PC-4	PC-5	PC-6	PC-7	PC-8	PC-9	PC-10	PC-11	PC-12	PC-13	PC-14	PC-15
Calibration	59.71	78.70	86.09	91.67	93.87	95.37	96.39	97.22	97.66	98.03	98.35	98.58	98.76	98.93	99.07
Validation	58.87	76.71	83.23	88.30	90.51	91.92	92.55	93.71	94.33	94.71	95.14	95.56	95.61	95.99	96.73

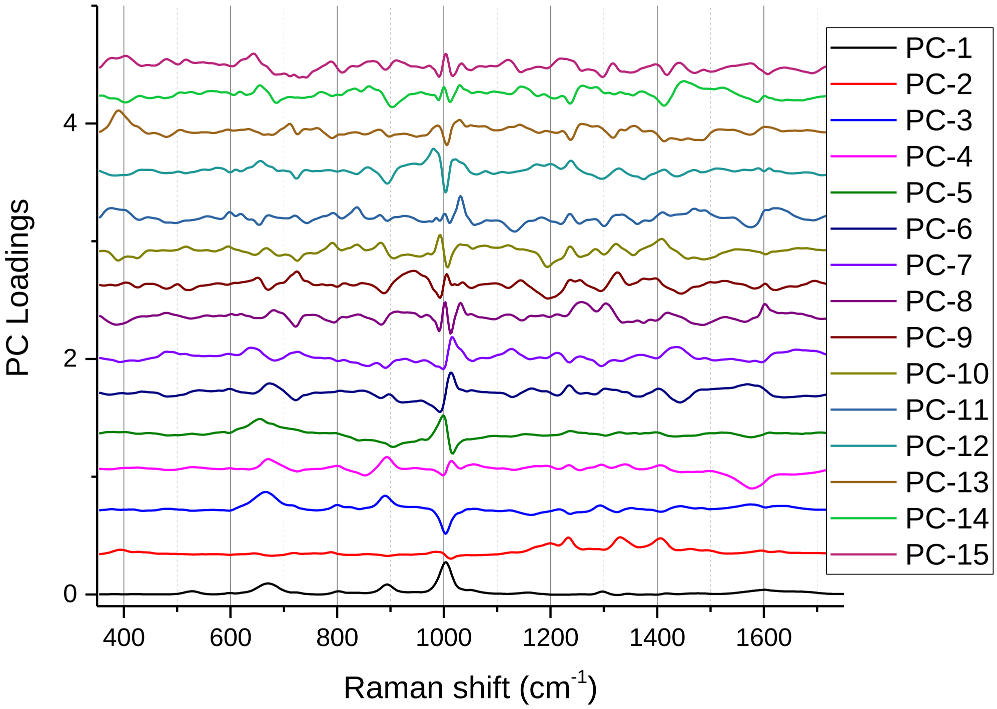

Figure S9. Loading plots for the 15 PCs in the PCA. The data were offset for clarity.

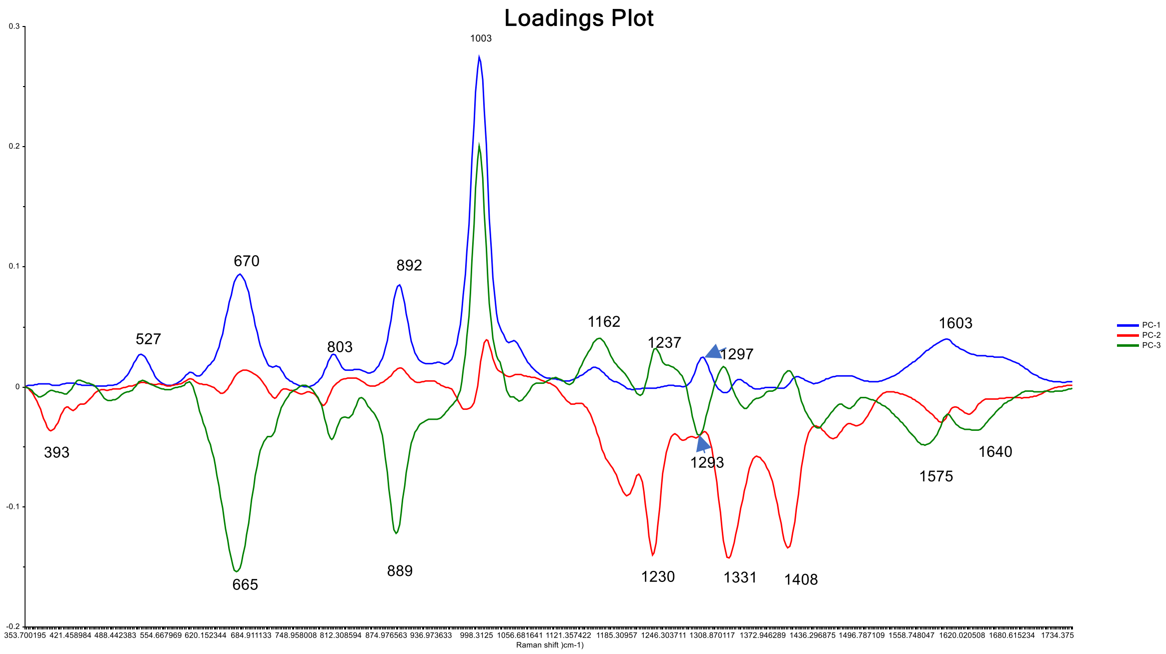

Figure S10. Detailed plot of the loading for the first 3 PCs in the PCA highlighting the major vibration bands contributing to the model.

Table S2. Discrimination parameters for the supervised LDA-PCA using the linear, quadratic and Mahalanobis discrimination functions and 2-15 PCs

No of comp	Quadr Function					Mahalanobis Function					Linear Function				
	FP	FN	Sens.	Spec.	Accuracy	FP	FN	Sens.	Spec.	Accuracy	FP	FN	Sens.	Spec.	Accuracy
2	6	19	0.62	0.86	0.73	17	13	0.74	0.61	0.68	14	13	0.74	0.68	0.71
3	6	12	0.76	0.86	0.81	12	6	0.88	0.73	0.81	17	13	0.74	0.61	0.68
4	8	10	0.8	0.82	0.81	8	8	0.84	0.82	0.83	11	7	0.86	0.75	0.81
5	8	8	0.84	0.82	0.83	3	10	0.8	0.93	0.86	12	8	0.84	0.73	0.79
6	9	7	0.86	0.80	0.83	3	9	0.82	0.93	0.87	9	5	0.90	0.80	0.85
7	8	4	0.92	0.82	0.87	4	7	0.86	0.91	0.88	9	5	0.90	0.80	0.85
8	4	5	0.9	0.91	0.90	3	5	0.9	0.93	0.91	6	3	0.94	0.86	0.90
9	2	3	0.94	0.95	0.95	1	4	0.92	0.98	0.95	3	3	0.94	0.93	0.94
10	2	3	0.94	0.95	0.95	1	5	0.9	0.98	0.94	1	2	0.96	0.98	0.97
11	3	3	0.94	0.93	0.94	1	6	0.88	0.98	0.93	1	3	0.94	0.98	0.96
12	1	3	0.94	0.98	0.96	0	6	0.88	1.00	0.94	1	3	0.94	0.98	0.96
13	1	3	0.94	0.98	0.96	1	4	0.92	0.98	0.95	1	3	0.94	0.98	0.96
14	0	1	0.98	1.00	0.99	0	3	0.94	1.00	0.97	0	1	0.98	1.00	0.99
15	0	0	1	1.00	1.00	0	0	1	1.00	1.00	0	1	0.98	1.00	0.99

FP-false positive; FN-false negative; TN-true negative; TP-true positive

Accuracy= TP+TN/(TP+FN+FP+FN); Sensitivity= TP/(TP+FN); Specificity= TN/(TN+FP)

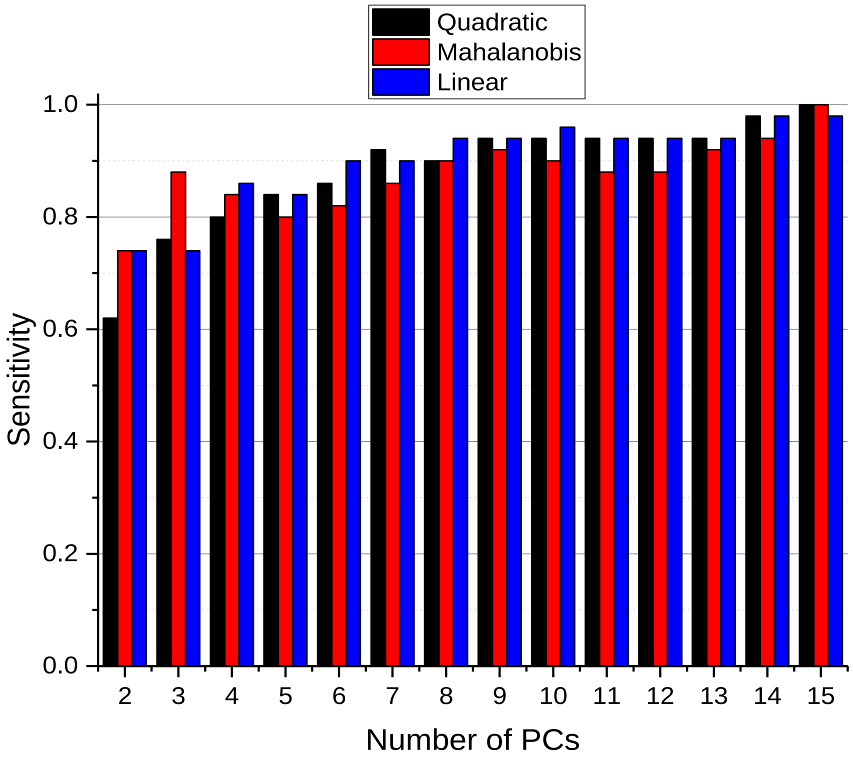

Figure S11. Sensitivity of discrimination between controls and RCC cases with different numbers of PC components and 3 discrimination functions: quadratic (black), Mahalanobis (red), and linear (blue).

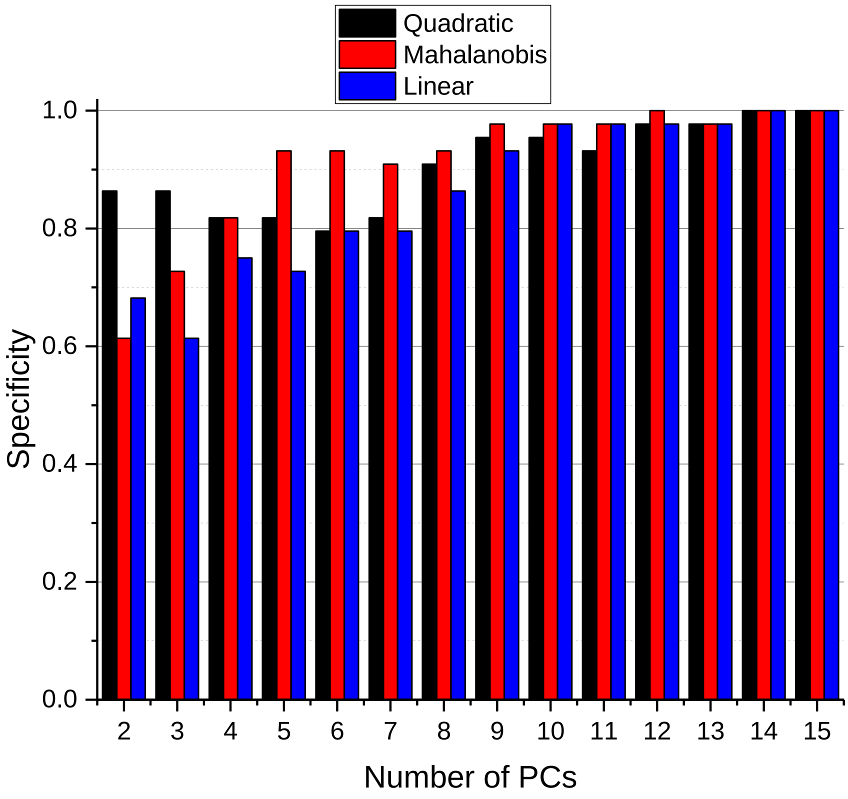

Figure S12. Specificity of discrimination between controls and RCC cases with different numbers of PC components and 3 discrimination functions: quadratic (black), Mahalanobis (red), and linear (blue).

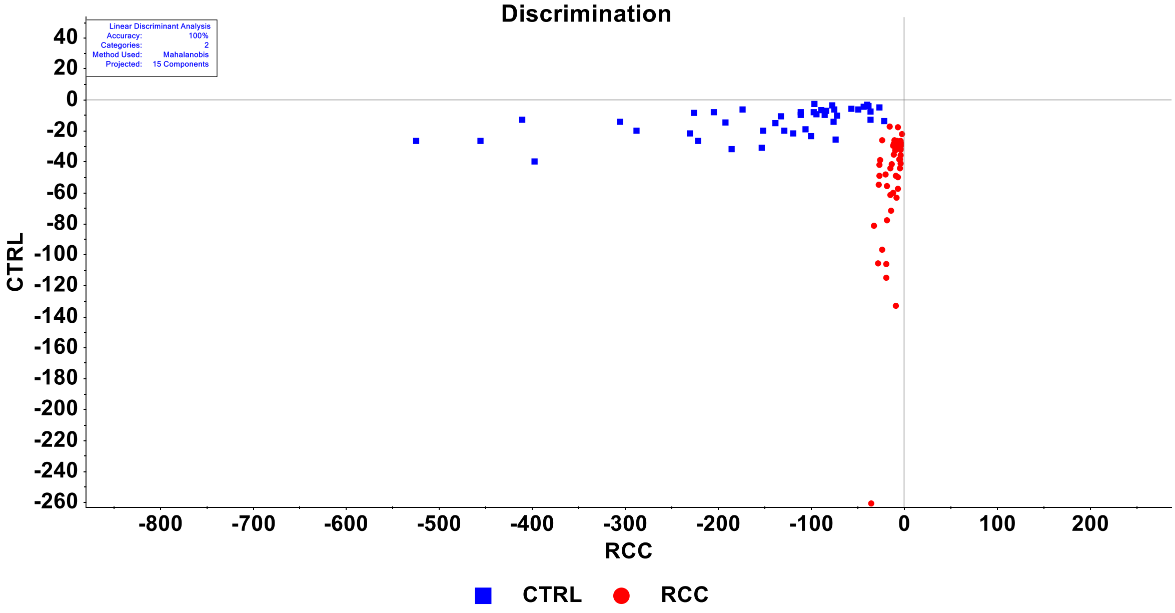

Figure S13. Discrimination plot between the RCC and CTRL cases obtained using the PCA-LDA with 15 PCs and the Mahalanobis discrimination function

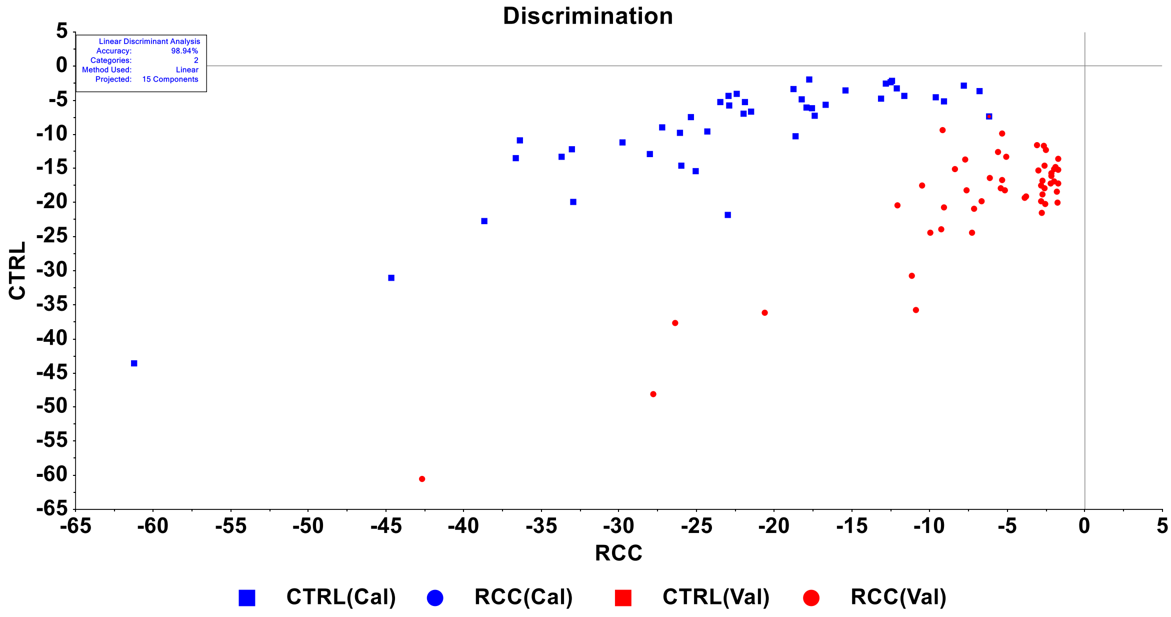

Figure S14. Discrimination plot between the RCC and CTRL cases obtained using the PCA-LDA with 15 PCs and the linear discrimination function. The red circle in a blue square represents the single CTRL sample classified as RCC.

Table S3. Confusion matrix for LDA-PCA discrimination of RCC stages using the first 7 PCs and a quadratic discrimination function.

	CTRL	Stage 1	Stage 2	Stage 3	Predicted
**CTRL**	**40**	2	1	0	**43**
**Stage 1**	4	**27**	3	3	**37**
**Stage 2**	0	3	**7**	0	**10**
**Stage 3**	0	0	0	**4**	**4**
**Actual**	**44**	**32**	**11**	**7**	

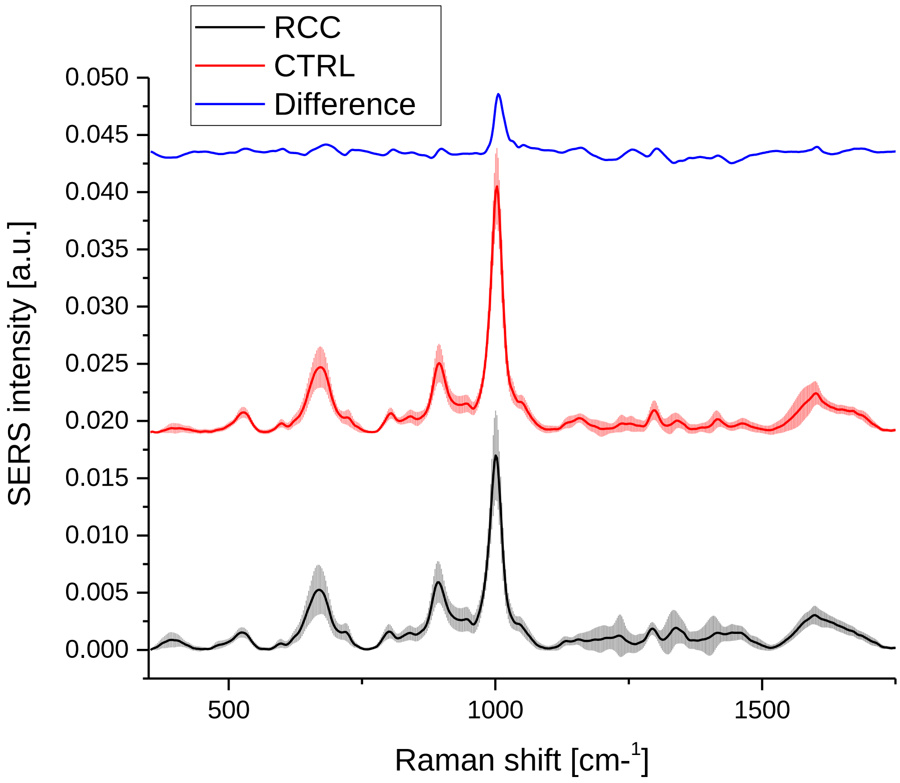

Figure S15. Mean SERS intensities for the CTRL and RCC cases and the difference spectrum (CTRL-RCC). The dashed areas represent the standard deviations.

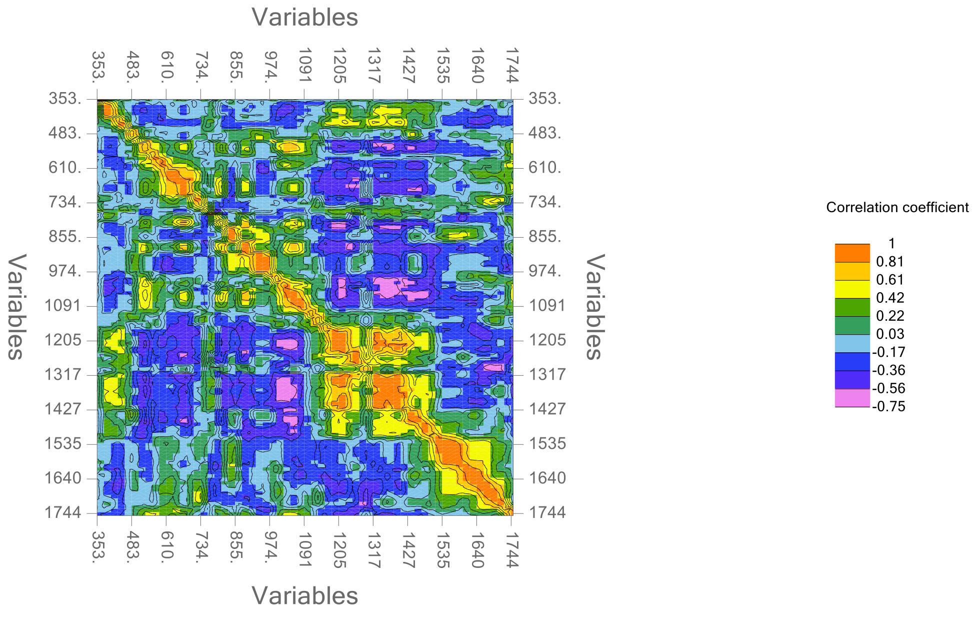

Figure S16. Contour plot of the correlation matrix for the SERS intensities recorded at different wavenumbers (Raman shifts) based on the SERS spectra mediated over all samples and area normalized. The coefficient of correlation is represented by different colors according to the legend figured on the right of the matrix plot.

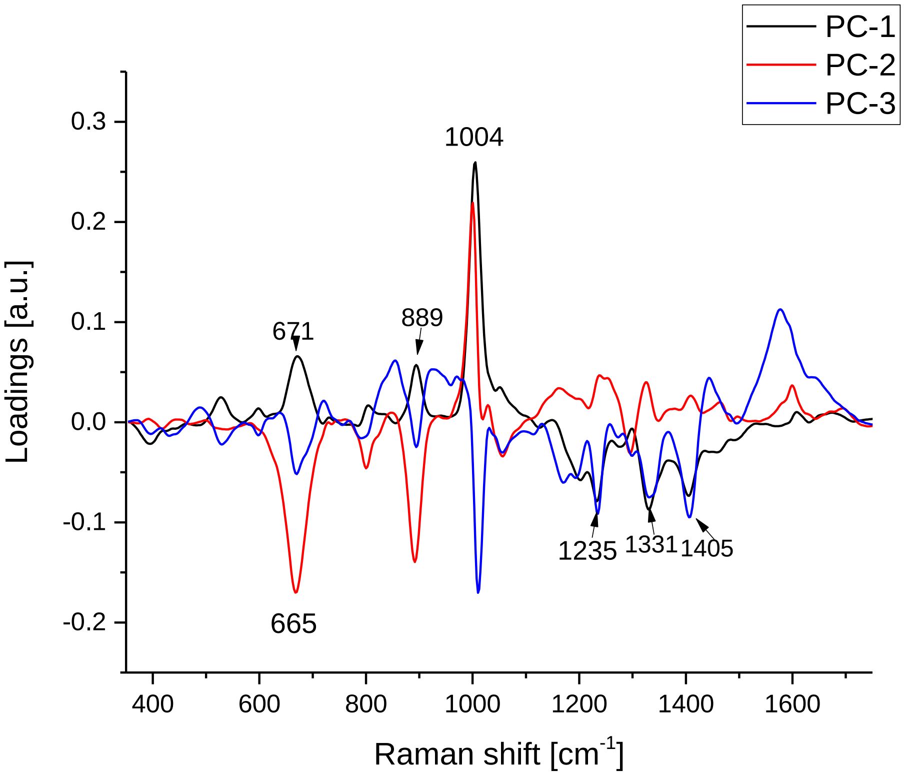

Figure S17. Loading plots for the first 3 PC in the PCA for the area normalized data indicating the major vibration contributing to the model

Table S4. Pearson's coefficient calculated from the Student's t-tests between the RCC and CTRL groups based on their scores in PCA for area-normalized spectra.

No of PC	1	2	3	4	5	6	7	8	9	10	11	12	13
p	<0.001	0.028	0.007	0.11	<0.001	0.011	0.002	0.202	0.207	0.278	0.18	0.038	0.498

Table S5. Confusion matrix for the classification of the samples based on stages using VSM and a linear kernel

	**CTRL**	**Stage 1**	**Stage 2**	**Stage 3**	**Predicted**
**CTRL**	44	0	0	0	**44**
**Stage 1**	0	32	11	7	**50**
**Stage 2**	0	0	0	0	**0**
**Srage3**	0	0	0	0	**0**
**Actual**	**44**	**32**	**11**	**7**	

Table S6. Demographic data and tumor-related information of the renal cell carcinoma patients enrolled in the study.

Number	Age (years)	Sex	Tumor histology	TNM[1]	Stage	ISUP[2] grade	Intratumoral necrosis	Intratumoral bleeding
1	66	M	Clear cell carcinoma	T1bN0M0	1	3	Yes	No
2	76	M	Clear cell carcinoma	T3aN0M0	3	4	Yes	Yes
3	73	M	Clear cell carcinoma	T1aN0M0	1	1	No	No
4	44	M	Clear cell carcinoma	T1aN0M0	1	2	No	Yes
5	73	M	Clear cell carcinoma	T3aN0M0	3	2	No	Yes
6	62	M	Clear cell carcinoma	T1bN0M0	1	2	Yes	Yes
7	56	M	Clear cell carcinoma	T1bN0M0	1	2	No	Yes
8	64	M	Clear cell carcinoma	T1aN0M0	1	1	No	No
9	77	M	Clear cell carcinoma	T2aN0M0	2	2	No	No
10	67	M	Clear cell carcinoma	T1bN0M0	1	2	No	No
11	60	M	Clear cell carcinoma	T1aN0M0	1	2	No	Yes
12	59	M	Clear cell carcinoma	T1aN0M0	1	2	No	Yes
13	72	M	Clear cell carcinoma	T2aN0M0	2	3	Yes	Yes
14	67	M	Clear cell carcinoma	T3aN0M0	3	4	Yes	Yes
15	75	M	Clear cell carcinoma	T2aN0M0	2	2	No	Yes
16	47	M	Clear cell carcinoma	T2aN0M0	2	1	Yes	Yes
17	61	M	Clear cell carcinoma	T2aN0M0	2	2	No	Yes
18	55	M	Clear cell carcinoma	T1aN0M0	1	2	No	Yes
19	61	M	Clear cell carcinoma	T1aN0M0	1	2	No	No
20	64	M	Clear cell carcinoma	T3aN0M0	3	2	Yes	Yes
21	54	M	Clear cell carcinoma	T1aN0M0	1	2	No	Yes
22	62	M	Clear cell carcinoma	T1bN0M0	1	4	Yes	Yes

23	53	M	Clear cell carcinoma	T2aN0M0	2	2	No	Yes
24	60	M	Clear cell carcinoma	T2aN0M0	2	3	No	Yes
25	64	M	Clear cell carcinoma	T1bN0M0	1	1	Yes	Yes
26	60	M	Clear cell carcinoma	T2aN0M0	2	1	No	Yes
27	44	M	Clear cell carcinoma	T1bN0M0	1	3	No	No
28	66	M	Clear cell carcinoma	T1aN0M0	1	1	No	Yes
29	38	M	Clear cell carcinoma	T1aN0M0	1	1	No	No
30	59	M	Clear cell carcinoma	T3aN0M0	3	2	Yes	Yes
31	69	M	Clear cell carcinoma	T1bN0M0	1	2	No	Yes
32	74	M	Clear cell carcinoma	T1bN0M0	1	2	Yes	Yes
33	76	M	Clear cell carcinoma	T2aN0M0	2	2	Yes	Yes
34	72	M	Clear cell carcinoma	T1aN0M0	1	2	No	Yes
35	70	M	Clear cell carcinoma	T1bN0M0	1	1	No	Yes
36	69	M	Clear cell carcinoma	T1bN0M0	1	1	Yes	Yes
37	57	M	Clear cell carcinoma	T1aN0M0	1	2	No	No
38	56	M	Clear cell carcinoma	T1bN0M0	1	1	No	Yes
39	63	M	Clear cell carcinoma	T2aN0M0	2	2	No	Yes
40	78	M	Clear cell carcinoma	T1aN0M0	1	2	No	Yes
41	64	M	Clear cell carcinoma	T3aN0M0	3	4	Yes	Yes
42	65	M	Clear cell carcinoma	T1aN0M0	1	2	No	Yes
43	56	M	Clear cell carcinoma	T3aN0M0	3	2	Yes	Yes
44	70	M	Clear cell carcinoma	T1aN0M0	1	2	No	Yes
45	50	M	Clear cell carcinoma	T1aN0M0	1	2	No	No
46	69	M	Clear cell carcinoma	T1aN0M0	1	2	No	Yes
47	41	M	Clear cell carcinoma	T1aN0M0	1	1	No	No

48	54	M	Clear cell carcinoma	T1bN0M0	1	4	Yes	Yes
49	48	M	Clear cell carcinoma	T1aN0M0	1	2	No	Yes
50	68	M	Clear cell carcinoma	T2aN0M0	2	2	No	Yes

1-Tumor Node Metastases
2-International Society of Urologic Pathologists

Table S7. Demographic data of control patients.

Number	Age (Years)	Sex
1	40	M
2	57	M
3	64	M
4	79	M
5	62	M
6	48	M
7	68	M
8	68	M
9	74	M
10	60	M
11	48	M
12	38	M
13	84	M
14	53	M
15	73	M
16	59	M
17	66	M
18	19	M
19	31	M
20	72	M
21	74	M
22	19	M
23	83	M
24	45	M
25	73	M
26	54	M
27	65	M
28	82	M
29	68	M
30	50	M
31	60	M
32	74	M
33	73	M
34	88	M
35	62	M
36	52	M
37	70	M

38	66	M
39	62	M
40	26	M
41	64	M
42	59	M
43	51	M
44	53	M

Table S8. Age statistics for the RCC patients and the controls enrolled in this study

	N total	Mean	Standard Deviation	Minimum	Median	Maximum
Controls	44	59.91	16.4	19	62	88
RCC Patients	50	62.16	9.8	38	63.5	78
